# A Rhodium–Pentane Sigma‐Alkane Complex: Characterization in the Solid State by Experimental and Computational Techniques

**DOI:** 10.1002/anie.201511269

**Published:** 2016-02-16

**Authors:** F. Mark Chadwick, Nicholas H. Rees, Andrew S. Weller, Tobias Krämer, Marcella Iannuzzi, Stuart A. Macgregor

**Affiliations:** ^1^ Department of ChemistryUniversity of OxfordMansfield RoadOxfordOX1 3TAUK; ^2^ Institute of Chemical SciencesHeriot-Watt UniversityEdinburghEH14 4ASUK; ^3^ Physical-Chemistry InstituteUniversity of ZürichWinterthurerstrasse 1908057ZürichSwitzerland

**Keywords:** alkanes, C−H activation, computation, rhodium, sigma complexes

## Abstract

The pentane σ‐complex [Rh{Cy_2_P(CH_2_CH_2_)PCy_2_}(η^2^:η^2^‐C_5_H_12_)][BAr^F^_4_] is synthesized by a solid/gas single‐crystal to single‐crystal transformation by addition of H_2_ to a precursor 1,3‐pentadiene complex. Characterization by low temperature single‐crystal X‐ray diffraction (150 K) and SSNMR spectroscopy (158 K) reveals coordination through two Rh⋅⋅⋅H−C interactions in the 2,4‐positions of the linear alkane. Periodic DFT calculations and molecular dynamics on the structure in the solid state provide insight into the experimentally observed Rh⋅⋅⋅H−C interaction, the extended environment in the crystal lattice and a temperature‐dependent pentane rearrangement implicated by the SSNMR data.

The synthesis of σ‐alkane complexes,^1^ in which an alkane interacts with a metal center through 3‐center–2‐electron M⋅⋅⋅H−C bonds,^2^ is of significant interest in terms of the development of new synthetic methodologies for C−H activation processes, especially the controlled functionalization of fossil‐derived hydrocarbons.^3^, ^4^ Owing to the strong, non‐polar, C−H bond, and steric interactions from proximal alkyl groups, alkanes are poor ligands,^5^ meaning that solvent or other Lewis bases can compete for metal coordination. Direct observation of alkane complexes in solution thus requires low temperatures (typically 130–190 K) and spectroscopic techniques, such as time‐resolved infrared spectroscopy (TRIR)^6^ or in situ NMR spectroscopy,^7^ and is often (although not exclusively^8^) based on reversible ligand photo‐ejection. When this relative instability is coupled with the less than 100 % efficiency often associated with the generation of σ‐alkane complexes, the production of single‐crystalline material suitable for detailed structural characterization is very challenging. Nevertheless, initially serendipitous, single‐crystal X‐ray diffraction studies have shown alkane C−H bonds in close approach with metal centers (Fe^2+^,^9^ U^3+^,^10^ K^+[11]^), in which host–guest interactions with alkane ligands are suggested to play an important role. We recently reported an alternative approach for the targeted generation of a σ‐alkane complex by solid/gas single‐crystal to single‐crystal transformations^12^ that converts [Rh{R_2_P(CH_2_CH_2_)PR_2_}(η^2^:η^2^‐NBD)][BAr^F^_4_] [R=^i^Bu, Cy; NBD=norbornadiene, Ar^F^=3,5‐(CF_3_)_2_(C_6_H_3_)] to [Rh{R_2_P(CH_2_CH_2_)PR_2_}(η^2^:η^2^‐NBA)][BAr^F^_4_] ([**1**][BAr^F^_4_]; NBA=norbornane, R=^i^Bu, **1 a**, Cy, **1 b**; Scheme 1).^13^, ^14^ For R=Cy, this produces a remarkably stable (months at 298 K) σ‐alkane complex that allows for full characterization by single‐crystal X‐ray diffraction and solid‐state NMR spectroscopy, supported by DFT calculations on the isolated cation. For complex [**1 a**][BAr^F^_4_], NBA loss (at 298 K) forms the [BAr^F^_4_]‐coordinated zwitterion. Although the underlying factors that control the relative stabilities of [**1 b**][BAr^F^_4_] and [**1 a**][BAr^F^_4_] in the solid state are currently unresolved, in both species an octahedral arrangement of [BAr^F^_4_]^−^ anions surrounds each cation, providing a cavity that supports the synthesis of the alkane adduct in the solid state.

**Scheme 1:**
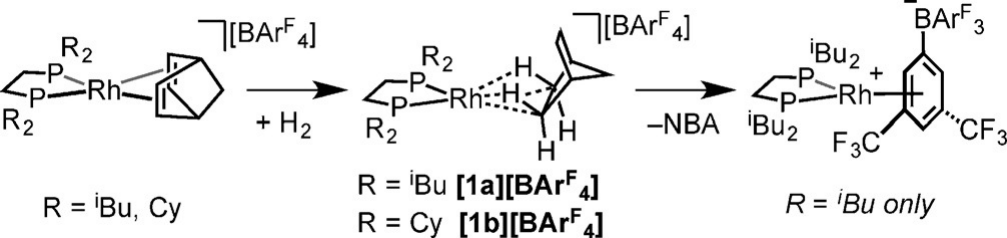
Synthesis of complexes **1** by a solid/gas single‐crystal to single‐crystal transformation.

The functionalization by C−H activation (via intermediate σ‐complexes) of simple, light, but low‐value hydrocarbons, such as butane and pentane, is important for their conversion into valuable products that can then enter the chemical feedstock chain.^15^ Although σ‐complexes of light alkanes have been characterized in situ by low temperature NMR and TRIR techniques,^16^ published examples that have been structurally characterized are highly disordered (heptane‐Fe‐porphyrin),^9^ or characterized as being mainly electrostatic in nature (K^+^/alkane).^11^ Powder neutron diffraction studies at 4–8 K show the binding of ethane or propane with an Fe center in a metal–organic framework.^17^ We now demonstrate that a well‐defined σ‐alkane complex of a linear light alkane (pentane) at a late transition metal (rhodium) can be generated by a simple solid/gas reaction, and structurally characterized in the solid state using single‐crystal X‐ray diffraction, solid‐state NMR (SSNMR), and computational techniques (Scheme 2). This provides detailed structural metrics for the coordination of a linear, light, alkane at a metal center well known for promoting C−H activation.^4^

**Scheme 2:**
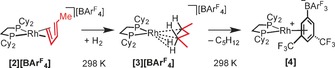
Synthesis of complex [**3**][BAr^F^] by a solid/gas single‐crystal to single‐crystal transformation, and its onward reactivity.

To produce the target Rh‐pentane complex, the pentadiene precursor [Rh{Cy_2_P(CH_2_CH_2_)PCy_2_}(η^2^:η^2^‐C_5_H_8_)][BAr^F^_4_] ([**2**][BAr^F^_4_]) was synthesized.^18^ A single‐crystal X‐ray diffraction study (Figure 1 A) showed disorder in both the Cy groups and the pentadiene fragment (*P*‐1, *Z*=2, *V=*3244(1) Å^3^, *R*(2σ)=7.2 %), with the latter showing two orientations (approximately 1:1 ratio) of the alkene fragment, discriminated by the methyl group pointing in opposite directions (that is, towards P1 or P2). Nevertheless, C−C/C=C alternations in bond lengths of the diene can be clearly discriminated, while an orientation is adopted as expected for coordination through the π‐face (angle between the planes defined by Rh1‐P1‐P2 and C6 to C10 being 102.3°). A ^31^P{^1^H} SSNMR spectrum of [**2**][BAr^F^_4_] shows four environments (298 K, *J*(RhP) ≈145 Hz; Supporting Information, Figure S1), reflecting the lack of crystallographic symmetry in the molecule and that the two orientations of the diene do not interchange in the solid state on the SSNMR timescale (that is, static disorder). Likewise, the ^13^C{^1^H} SSNMR spectrum shows 7 alkene resonances between *δ*=104 and 60 ppm, with the lowest field signal assigned to a 1+1 coincidence. No high field signals, which would signal Rh⋅⋅⋅H−C or C−H⋅⋅⋅aryl interactions,^13^, ^19^ were observed in the ^1^H NMR projection from a frequency‐switched Lee–Goldburg (FSLG) SSNMR experiment (Figure S2).

**Figure 1:**
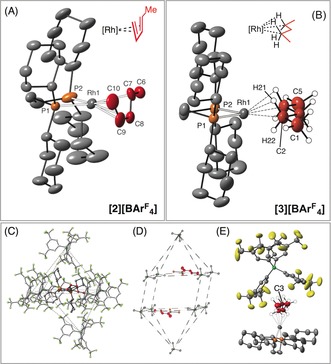
A) Solid‐state structure of the cationic portion of [**2**][BAr^F^_4_], displacement ellipsoids are shown at 50 %, only one disorder component is shown. Hydrogen atoms are not shown. Selected distances (Å) and angles (°): Rh1‐P1, 2.272(1); Rh1‐P2, 2.264(1); Rh1‐C7, 2.33(1); Rh1‐C8, 2.26(1); Rh1‐C9, 2.25(2); Rh1‐C10, 2.32(1); C7‐C6, 1.529(8); C7‐C8, 1.386(9); C8‐C9, 1.540(8); C9‐C10, 1.272(9); angle between planes defined by Rh1‐P1‐P2/C6 to C10=102.3°. B) Solid‐state structure of the cationic portion of [**3**][BAr^F^_4_], displacement ellipsoids are shown at 50 %, selected hydrogen atoms shown; Rh1‐P1, 2.197(1); Rh1‐P2, 2.196(1); Rh1‐C2, 2.514(4); Rh1‐C4, 2.522(5); C1‐C2, 1.519(7); C2‐C3, 1.534(7); C3‐C4, 1.533(7); C4‐C5, 1.537(7); C2‐H21, 0.87(5); C2‐H22, 1.07(5); C4‐H41, 0.83(5); C4‐H42, 1.02(5); Rh1‐H21, 2.24(5); Rh1‐H41, 2.21(5); C2‐C3‐C4, 110.4(4); C1‐C2‐C3, 113.8(4); C3‐C4‐C5, 114.3(4); angle between planes defined by Rh1‐P1‐P2/C1 to C5=9.5°. C) Local anion environment surrounding one cation in [**2**][BAr^F^_4_]; D) Extended lattice environment for [**3**][BAr^F^_4_] highlighting two cations encapsulated in a bicapped square‐prism of anions (aryl groups and phosphine groups removed); E) Relationship between [**3**]^**+**^ and a single [BAr^F^_4_]^−^ anion. Shortest distance between H‐atoms on C3 and the centroids of the two aryl rings=2.72, 3.01 Å.

Addition of H_2_ to crystalline, burgundy‐colored, [**2**][BAr^F^_4_] (2 atm, 2 min, 298 K), and rapid transfer to a pre‐cooled X‐ray diffractometer (150 K), resulted in single‐crystalline material of the new, 16‐electron, pentane complex [Rh{Cy_2_P(CH_2_CH_2_)PCy_2_}(η^2^:η^2^‐C_5_H_12_)][BAr^F^_4_] ([**3**][BAr^F^_4_]; Figure 1 B). Remarkably, the refined structure of [**3**][BAr^F^_4_] was significantly improved compared to [**2**][BAr^F^_4_], and there was no disorder present (*P*‐1, *Z*=2, *V=*3259(1) Å^3^, *R*(2σ)=5.6 %). Hydrogenation of the pentadiene to form pentane was indicated by three significant structural differences: i) A change in orientation on replacement of the Rh⋅⋅⋅π‐alkene interaction with σ−Rh⋅⋅⋅H−C so that the Rh1‐P1‐P2/C1 to C5 inter‐plane angle=9.5° in [**3**][BAr^F^_4_]; ii) The C−C lengths indicate C−C single bonds only (1.519(7)–1.537(7) Å); iii) Rh−P lengths in [**3**][BAr^F^_4_] are considerably shorter than in [**2**][BAr^F^_4_], reflecting the low *trans*‐influence alkane ligand (2.197(1), 2.196(1) Å compared to 2.272(1), 2.264(1) Å). The pentane ligand binds with the metal as the 2,4‐isomer rather than an alternative motif, for example, 1,3, 1,4, or 1,5. The Rh⋅⋅⋅C distance (2.514(4) and 2.522(5) Å) in [**3**][BAr^F^_4_] are well within the combined van der Waals radii of Rh and C−H (4.44 Å),^20^ but are longer than observed for [**1 b**][BAr^F^_4_] (2.389(3), 2.400(3) Å), suggesting a weaker interaction. However, the Rh−P distances are relatively insensitive to this difference, being the same within error between the two complexes. The structural refinement was of sufficient quality to determine the location of the C−H hydrogen atoms. However, within the precision of the X‐ray diffraction experiment, the C−H (for example, C2‐H21, 0.87(5); C2‐H22, 1.07(5) Å) or Rh⋅⋅⋅H distances (Rh1‐H21, 2.24(5), Rh1‐H22 2.28(5) Å) are not statistically different, meaning that the experimental data cannot discriminate between η^2^‐HC or η^3^‐H_2_C^21^ binding modes.

Periodic DFT calculations^18^ employing the PBE‐D3 functional on the extended solid‐state structure of [**3**][BAr^F^_4_] reproduce the metrics associated with the heavy atoms extremely well (see Figure 2 A for an overlay with the unit cell of the experimental structure). The computed structure also permits a more detailed discussion of the rhodium–pentane interaction (Figure 3), and shows an average computed Rh⋅⋅⋅H21/Rh⋅⋅⋅H41 distance of 2.02 Å, significantly shorter than Rh⋅⋅⋅H22/H42 (2.31 Å); in addition the C2‐H21/C4‐H41 distance is 1.14 Å, somewhat longer than C2‐H22/C4‐H42 (1.11 Å). These data suggest a bis‐η^2^‐HC binding mode similar to that observed for [**1**][BAr^F^_4_]. This was supported by an Atoms‐in‐Molecules (AIM) study of the computed electron density of the [**3**]^**+**^ cation in which bond critical points (BCPs) were only located between Rh and each of H21 and H41 (*ρ*_ave_=0.047 au), with no BCPs between Rh and either H22 or H42. Moreover, reduced electron densities are associated with the C2‐H21/C4‐H41 BCPs (*ρ*_ave_=0.245 au), compared to the C2‐H22/C4‐H42 BCPs (*ρ*_ave_=0.265 au). These computed geometric and AIM data also suggest the rhodium–pentane interaction in [**3**][BAr^F^_4_] is somewhat weaker than the Rh‐NBA interaction in [**1 b**][BAr^F^_4_], and this was confirmed by an NBO analysis in which the overall degree of C−H→Rh σ‐donation and Rh→C−H π‐back donation is approximately halved in the pentane complex (Table S5).

**Figure 2:**
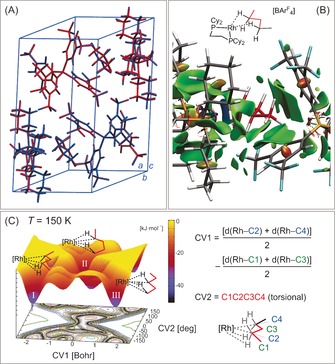
A) DFT‐optimized geometry of the unit cell contents of [**3**][BAr^F^_4_] (blue) overlaid onto the crystal structure (red). B) Non‐covalent interaction plot within one [**3**][BArF_4_] molecular unit (promolecular density). Isosurfaces plotted with a reduced density gradient isovalue of *s*=0.35 a.u. C) Free Energy Surface (FES) from metadynamics sampling at *T=*150 K and definition of collective variables (CV).

**Figure 3:**
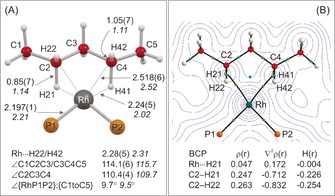
A) Comparison of experimental (plain text) and computational (italics) structural metrics, the latter derived from periodic calculations on the extended solid‐sate structure using the PBE‐D3 functional. Average distances (Å) and angles (°) are provided. B) Contour plot of the total electron density in the {RhH21H41} plane showing bond critical points (BCP) in green and ring critical point (RCP) in blue (selected critical points and bond paths cloaked for clarity). Calculated parameters (au) are given for selected BCPs.

The [BAr^F^_4_]^−^ anions in the lattice of [**2**][BAr^F^_4_] are arranged as a distorted bicapped prism (Figure 1 C) that encompasses two cations. This is in contrast to [**1 b**][BAr^F^_4_], and its NBD precursor, in which the [BAr^F^_4_]^−^ anions form a distorted octahedron, although both provide a well‐defined cavity. Upon hydrogenation to form [**3**][BAr^F^_4_], there was no significant change in the arrangement of the [BAr^F^_4_]^−^ anions, and the unit cell volume barely changed (3244(1) versus 3259(1) Å^3^), reflecting the retention of crystallinity.

Previously, we have shown that the intrinsic alkane binding energy computed in isolated [**1 a**]^**+**^, [**1 b**]^**+**^, and related molecular cations bears little correlation to the observed longevity of the alkane complex in the solid state.^13^ The extended environment is therefore crucial in stabilizing these σ‐alkane complexes, and some components of this are evident in the non‐covalent interaction (NCI) plot (Figure 2 B). This highlights broad regions of green (weakly stabilizing) van der Waals interactions that enfold the pentane moiety between two aryl arms of the [BAr^F^_4_]^−^ anion. In addition, the green disks located along several of the C−F⋅⋅⋅H−C vectors are indicative of weak hydrogen bonding, the cumulative effect of which will also be to enhance the solid‐state stability.

As with the solid‐state structure, the low‐temperature (223 K) ^31^P{^1^H} SSNMR spectrum of [**3**][BAr^F^_4_] again presents a simpler analysis than [**2**][BAr^F^_4_], as the static disorder in the pentadiene complex is no longer present (Figure S4). The principal component is observed as two sets of doublets centered at *δ*=107.6 and 104.1 ppm (*J*(RhP)=235, 233 Hz). The ^103^Rh−^31^P coupling constants are significantly larger than in [**2**][BAr^F^_4_], consistent with the weaker *trans*‐influence alkane. Also apparent is a broad signal at *δ*=90 ppm (approximately 30 %) that is assigned to the product of pentane loss and coordination of [BAr^F^_4_]^−^: [Rh{Cy_2_P(CH_2_CH_2_)PCy_2_}{η^6^‐(C_6_H_3_)(CF_3_)_2_BAr^F^_3_}] **4**.^13^, ^14^, ^22^ At 223 K the spectra remain unchanged overnight, but on warming to 298 K (12 hrs), **4** grows in at the expense of [**3**][BAr^F^_4_], demonstrating that the pentane complex is thermally unstable (Figure S8). This instability can also be followed by single‐crystal X‐ray diffraction, which shows a relatively rapid (15 minutes at 298 K) loss of high‐angle data on warming (Figure S13). Interestingly, [**1 b**][BAr^F^_4_] (which has the same {RhL_2_}^+^ fragment) does not decompose in the solid state.

The ^13^C{^1^H} SSNMR (223 K, Figure S4) spectrum shows the disappearance of signals in the 100–60 ppm region, consistent with the hydrogenation of the diene. We have previously shown the utility of ^1^H/^13^C FSLG HETCOR SSNMR experiments combined with computed chemical shifts to identify resonances owing to the σ−Rh⋅⋅⋅H−C interactions and ring‐current‐affected chemical shifts in [**1 b**][BAr^F^_4_],^13^ following the use of similar techniques to identify agostic Ru⋅⋅⋅H−Si interactions in the solid state.^19^ Calculations using the periodic GIPAW methodology indicate that the σ−Rh⋅⋅⋅H−C interactions (C2/4) correspond to *δ*(^1^H)=−1.6/−2.5 ppm, and correlate to *δ*(^13^C)=7.3/9.9 ppm, respectively. The central methylene group (C3) experiences a ring current shift from proximal [BAr^F^_4_] aryl rings: *δ*(^1^H)=−2.4/−0.9 ppm, *δ*(^13^C)=40.1 ppm (Scheme 3). At 223 K, the ^13^C{^1^H} NMR spectrum shows somewhat broad signals in the aliphatic region and no clear high‐field correlations observed by FSLG HETCOR SSNMR experiments. Cooling to 158 K resolves the ^13^C{^1^H} NMR spectrum more clearly, so that sharper signals and strong correlations are now observed that match well with the computational model, and show the expected relative differences in chemical shifts (Figure S5–7). On temperature cycling between 223 K and 158 K, there are no significant changes in the ^31^P{^1^H} NMR spectra. Overall, these observations perhaps suggest a fluxional process may be occurring in the solid state at 223 K that is slowed at 158 K, so that the key C−H correlations and ^13^C signals that signal pentane coordination can be observed at this low temperature (Table S1). If [**3**][BAr^F^_4_] is left at 298 K for 7 days to form [**4**], the resulting ^1^H/^13^C FSLG HETCOR spectrum shows the absence of the correlations at *δ*(^13^C)=10 and 39 ppm that are assigned to the bound pentane at 158 K.

**Scheme 3:**
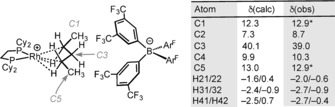
Selected calculated (GIPAW) and experimentally observed (FSLG‐HETCOR SSNMR) chemical shifts at 158 K for [**3**][BAr^F^_4_]. * Indicates a 1+1 coincidence in the experimental spectrum.^18^

To probe this fluxional process, we performed ab initio molecular dynamics on the structure of [**3**][BAr^F^_4_] in the solid state, where the metadynamics approach was applied to accelerate the exploration of conformational space. Two collective variables were selected to probe different coordination modes of the pentane ligand (Figure 2 C): CV1 discriminates between structures **I** and **III** (the 2,4‐ and 1,3‐isomers, respectively), while CV2 allows for further conformational flexibility, accessing structures such as **II** (a 1,4‐isomer). Metadynamic runs (≥120 ps) at 75, 150, and 300 K highlight changes in the amplitude of the structural fluctuations and allow the free energy surface (FES) to be plotted at each temperature. All three runs show variations in the coordination of pentane to Rh, with the initial 2,4‐isomer (CV1≈−1.8; CV2≈180.0°) and the 1,3‐isomer (CV1≈1.8; CV2≈180.0°) being stable states throughout; at 150 K, the 2,4‐isomer is more stable by approximately 4 kJ mol^−1^. The transition region linking these states corresponds to a variety of more flexible configurations in which pentane coordinates through one or two C−H bonds, suggesting facile interconversion with barriers of 15–25 kJ mol^−1^. The projection of the FES onto the subspace of the two CVs shows that increasing the temperature changes the relative stability of **I** and **III**, the trend favoring the 2,4‐isomer at 75 K, while the less rigid 1,3‐isomer dominates at 300 K (Figure S22). Similarly, the central region of the surface becomes more accessible at higher temperatures, reflecting a stabilization of the transition region owing to larger structural disorder. Overall, these results are consistent with a fluxional process between **I** and **III** at higher temperatures (223 K), which slows and shifts toward the 2,4‐isomer upon cooling to 158 K.

It is interesting to contrast the molecular dynamics FES with the outcome of static periodic calculations in which different pentane coordination modes are optimized at one of the Rh centers in the unit cell (Table S6). The computed electronic energies now indicate the 2,4‐isomer (**I**) lies 35 kJ mol^−1^ below the 1,3‐isomer (**III**). This reiterates the role of entropy in stabilizing the more conformationally flexible 1,3‐isomer, and how the *T*Δ*S* term makes this species more accessible at higher temperatures.

This proposed fluxional process is closely related to the chain‐walking events that have directly8b, 16c,16e and indirectly^23^ been shown to occur in transient, or only stable at low temperature, transition‐metal alkane σ‐complexes in solution (Scheme 4). With the pentane complex [**3**][BAr^F^_4_], similar processes could well be occurring in the solid state, connecting terminal (M⋅⋅⋅H_3_C) and internal (M⋅⋅⋅H_2_C) σ‐binding motifs, and the estimated barriers to this (15–25 kJ mol^−1^) are similar to experimentally measured values derived from solution‐measurements at very low temperature.8b A future challenge in this area is to explore if related complexes and processes are also possible for σ‐complexes with different alkane chain lengths, and whether these can exploited in the selective activation of C−H bonds.

**Scheme 4:**
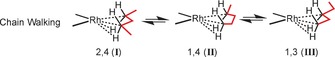
Pentane chain‐walking in the solid‐state. Abbreviated structure.

## Supporting information

As a service to our authors and readers, this journal provides supporting information supplied by the authors. Such materials are peer reviewed and may be re‐organized for online delivery, but are not copy‐edited or typeset. Technical support issues arising from supporting information (other than missing files) should be addressed to the authors.

Supplementary
