# Supplementary material for: A Rhodium–Pentane Sigma‐Alkane Complex: Characterization in the Solid State by Experimental and Computational Techniques
Source: Angew Chem Int Ed Engl. 2016 Feb 16;55(11):3677–81. doi: 10.1002/anie.201511269 (PMC4797708; doi:10.1002/anie.201511269)
Supplement: Supplementary file 1 — Supplementary [file ANIE-55-3677-s001.pdf]

Angewandte Chemie
Eine Zeitschrift der Gesellschaft Deutscher Chemiker

Supporting Information

A Rhodium–Pentane Sigma-Alkane Complex: Characterization in the Solid State by Experimental and Computational Techniques

F. Mark Chadwick[+], Nicholas H. Rees, Andrew S. Weller, Tobias Krämer[+], Marcella Iannuzzi, and Stuart A. Macgregor**

anie_201511269_sm_miscellaneous_information.pdf

Contents

1. General Considerations S2
2. Detailed Syntheses S5
3. Hydrogenation of [2][$BAr^F{}_4$] S9
4. Dissolution of [3][$BAr^F{}_4$] at room temperature S17
5. Dissolution of [3][$BAr^F{}_4$] at low temperature S18
6. Crystallographic information S20
7. Optimised geometries S22
8. AIM analysis S24
9. NBO analysis S25
10. Energies S26
11. Calculated $^1H/^{13}C$ NMR shifts S27
12. Further discussion of metadynamics S31
13. References S32

1. General Considerations

1.1 Practical Considerations

All manipulations (unless otherwise stated) were performed under an atmosphere of argon, using standard Schlenk techniques on a dual vacuum/inlet manifold or by employment of an MBraun glovebox. Glassware were dried in an oven at 130 °C overnight prior to use. Pentane, CH_2Cl_2 and hexane were dried using an MBraun SPS-800 solvent purification system and degassed by three freeze-pump-thaw cycles. CD_2Cl_2, and 1,2-$C_6H_4F_2$ were all dried by stirring over CaH_2 overnight before being vacuum distilled and subsequently degassed by three freeze-pump-thaw cycles.

Solution NMR data were collected on either a Brucker AVD 500 MHz or a Bruker Ascend 400 MHz spectrometer at room temperature unless otherwise stated. Residual protio solvent resonances were used as a reference for ^{1}H NMR spectra. ^{19}F and ^{31}P NMR spectra were referenced externally to CCl_3F and 85% H_3PO_4 respectively. All chemical shifts (δ) are quoted in ppm and coupling constants in Hz.

Solid state NMR (SSNMR) spectra (including two dimensional measurements) were obtained on a Bruker Avance III HD spectrometer equipped with a 9.4 Tesla magnet, operating at 399.9 MHz for ^{1}H and 100.6 MHz for ^{13}C using 4 mm O.D. rotors containing 70mg of sample and a MAS rate of 10 kHz. For ^{13}C CP/MAS a sequence with a variable X-amplitude spin-lock pulse[1] and spinal64 proton decoupling was used. 3400 transients were acquired using a contact time of 3 ms, an acquisition time of 25 ms (2048 data points zero filled to 24 K) and a recycle delay of 2 s. All ^{13}C spectra were referenced to adamantane (the upfield methine resonance was taken to be at δ = 29.5 ppm[2] on a scale where δ(TMS) = 0 as a secondary reference. For the FSLG-HETCOR,[3] 128 transients (2048 data points in F2) and 80 increments in F1 (zero filled to 4k x 1k) were acquired with a contact time 0.1 ms and a recycle delay of 5 s.

Electrospray ionisation mass spectrometry (ESI-MS) was carried out using a Bruker MicrOTOF-Q instrument directly connected to a modified Innovative Technology glovebox.[4] Typical acquisition parameters were used (sample flow rate: 4 µL min^{-1}, nebuliser gas pressure: 0.4 bar, drying gas: Argon at 333 K flowing at 4 L min^{-1}, capillary voltage: 4.5 kV, exit voltage: 60 V). The spectrometer was calibrated using a mixture of tetraalkyl ammonium bromides $[N(C_nH_{2n+1})_4]Br$ (n = 2-8, 12, 16 and 18). Samples were diluted to a concentration of 1 x 10^{-6} M in the appropriate solvent before running.

Single crystal X-ray diffraction data were collected as follows: a typical crystal was mounted on a MiTeGen Micromounts using perfluoropolyether oil and cooled rapidly to 150 K in a stream of nitrogen gas using an Oxford Cryosystems Cryostream unit.[5] Data were collected with an Agilent SuperNova diffractometer (Cu K_α radiation, λ = 1.54180 Å). Raw frame data were reduced CrysAlisPro.[6] The structures were solved using direct methods with SIR92[7] or SuperFlip[8] and refined using full-matrix least squares refinement on all F^2 data using the CRYSTALS program suite.[9,10] In general distances and angles were calculated using the full covariance matrix.

Elemental micro-analyses were carried out by Stephen Boyer at London Metropolitan University.

$Na[BAr^F_4]$ [Ar^F = (3,5-CF_3)-C_6H_3][11] and $[Rh(Cy_2PCH_2CH_2PCy_2)(\eta^2{:}\eta^2\text{-COD})_2][BAr^F_4]$[12] were prepared by literature procedures. All other chemicals were used as received from commercial

sources. $[Rh(Cy_2PCH_2CH_2PCy_2)(\eta^6\text{-}F_2C_6H_4)][BAr^F_4]$ is made from an adapted procedure of $[Rh(Cy_2PCH_2CH_2PCy_2)(\eta^6\text{-}FC_6H_5)][BAr^F_4]$.[12]

1.2 Computational Methods

1.2.1 Geometry optimisations

Periodic electronic structure calculations were carried out at the Kohn-Sham DFT level of theory, employing the Gaussian Plane Wave (GPW) formalism as implemented in the QUICKSTEP[14] module within the CP2K program suite (Version 2.7).[15] Molecularly optimized basis sets of double-ζ quality plus polarization in their shorter-range variant (DZVP-MOLOPT-SR-GTH)[16] were used on all atomic species (Rh, B, C, Si, P, F, H). The interaction between the core electrons and the valence shell (Rh: 17, B: 3, C: 4, Si: 4, P: 5, F: 7, H: 1 electrons) was described by Goedecker-Teter-Hutter (GTH) pseudo potentials.[17] The general gradient approximation (GGA) to the exchange-correlation functional according to Perdew-Burke-Ernzerhof (PBE)[18] was used in combination with Grimme's D3-correction for dispersion interactions.[19] The auxiliary plane wave basis set was truncated at a cutoff of 500 Ry. The maximum force convergence criterion was set to 10^{-4} Hartree·Bohr^{-1}, whilst default values were used for the remaining criteria. Optimized stationary points were characterized by analysis of their numerical second derivatives, with minima having only positive eigenvalues. The convergence criterion for the self-consistent field (SCF) accuracy was set to 10^{-7} E_h and 10^{-8} E_h for geometry optimisations and vibrational analysis, respectively. The Brillouin zone was sampled using the Γ-point. Initial coordinates of the 2,4-isomer were obtained from the experimental crystal structure, while other isomers were manually generated on one metal centre, maintaining the 2,4-binding mode at the other unit. Three-dimensional periodic boundary conditions (PBC) were applied in all cases in combination with fixed unit cell parameters (Å, deg) obtained from the experimental crystal structures (**[1b][BAr$^F_4$]**: *a* 19.0579 *b* 17.9828 *c* 19.5453 α 90.000 β 91.714 γ 90.000; **[2][BAr$^F_4$]**: *a* 12.8345 *b* 13.2654 *c* 20.0408 α 100.589 β 97.745 γ 100.760; **[3][BAr$^F_4$]**: *a* 13.0259 *b* 13.5044 *c* 19.1472 α 99.866 β 93.573 γ 99.433). Geometries were either fully relaxed (full optimization) or partially relaxed by allowing only hydrogen atoms to move during optimisation while constraining all other atoms to their crystal structure positions (X-ray structure refinement). Bond parameters obtained from a geometry optimisation of **[3][BAr$^F_4$]** using and extended 2×2×2 supercell were virtually identical those obtained using a single unit cell. The geometries of tetramethylsilane (TMS) was fully optimized following the same protocol as outlined above, but here a non-periodic cubic supercell of length 25 Å was employed.

1.2.2 Molecular dynamics

Born-Oppenheimer molecular dynamics[20] simulations have been performed at the same level of theory used for the geometry optimisations. The optimised structure has been taken as starting configuration and equilibrated by simulations in the canonical ensemble (NVT) increasing progressively the temperature up to 300 K. The equilibration has been extended for about 20 ps, without observing any relevant conformational change in the position of the two pentane units present in the simulation cell. In order to explore other regions of the accessible phase space, the metadynamics methodology has been used.[21, 22] In order to observe possible conformational changes within the cavity containing the pentane molecule, two collective variables were selected describing the coordination of one pentane unit with the metal centre and flexibility of its structure. These are the (average) difference of distances measured between the C atoms and Rh, defined as [d(C2-Rh)+d(C4-Rh)]/2-[d(C1-Rh)-d(C3-Rh)/2] and the torsion angle defined by four C atoms (C1C2C3C4). The metadynamics has been carried out adding Gaussian beads of penalty potential

every 30 fs along the molecular dynamics run. The size of the Gaussians have been kept rather small, i.e., height of 0.06 kcal mol^{-1} and width in the CV1 and CV2 dimensions of 0.09 Å and ~10°, respectively. This choice guarantees a gradual increase of the penalty potential and a thorough exploration of the smooth underlying free energy surface characterized by small energy barriers. The metadynamics has been performed starting from a snapshot of the equilibrated NVT trajectory. Three runs of about 130 ps have been carried out at the three different temperatures of 75 K, 150 K, and 300 K, in order to unravel changes in fluxionality of the structure by increasing the temperature.

1.2.3 Electronic structure analysis

All DFT calculations described in this section employed the GAUSSIAN 09 (revision D.01) suite of programs.[23] In order to generate electron densities suitable for further analysis, single-point calculations with the BP86 GGA functional[24] were performed on the geometries of the **[3]**$^+$ and **[1b]**$^+$ fragments taken from their respective CP2K-optimized crystal structures, without further relaxation. Stuttgart-Dresden (SDD)[25] relativistic effective core potentials (ECP) in combination with the associated basis sets were utilized to describe Rh and P, with a polarization functions added for P (ζ = 0.387).[26] The 6-31G(d,p) basis sets[27] were used on remaining atoms (B, C, and H). The topology of the electron density in was analysed by means of QTAIM (quantum theory of atoms in molecules),[28] as implemented in the AIMALL package.[29] Inner shell electrons on Rh and P modelled by ECPs were represented by core density functions (Extended Wavefunction format). Quantitative analysis of interactions between the alkane and $\{RhL_2\}^+$ fragments within the framework of second order perturbation theory was performed with NBO 5.9.[30] Non-covalent interactions within the **[3][BAr^F$_4$]** moiety were visualized with the NCIPLOT program, utilizing the promolecular densities approach.[31]

1.2.4 Calculation of magnetic shielding constants

Isotropic ^{13}C and ^1H magnetic shielding constants (σ_{iso}) were generated using the GIPAW method[32] as implemented in CASTEP 8.0.[33] Single-point calculations were performed on both the fully optimized and the refined crystal structure. The plane wave basis was truncated at 80 Ry, while the Brillouin zone was sampled using a Monkhorst-Pack grid[34] with k-point spacing of 0.04 $Å^{-1}$ (convergence of computed NMR parameters was evaluated with respect to the plane wave cutoff energy and k-point spacing, see Figures S15 and S16). The calculations were performed using the PBE GGA functional.[18] Ultra-soft pseudopotentials were generated on-the-fly to represent the core electron shells. Scalar-relativistic effects were incorporated through the zeroth-order regular approximation (ZORA). The default ultrafine SCF convergence threshold was used in the calculations (10^{-8} eV $atom^{-1}$). Computed ^{13}C isotropic shielding constants were converted into chemical shift values (δ) via a linear regression procedure.[35] To this end, calculated isotropic magnetic shielding tensors were plotted against selected experimental chemical shifts to yield a linear correlation plot. Data were collected for one asymmetric unit within the unit cell and averaged for the carbon atoms in the four $C_6H_3(CF_3)_2$ residues present in the BAr^F_4 counterion. Chemical shifts were then determined from the expression $\delta(^{13}C) = (\text{intercept} - \sigma_{iso}) / m$, where the intercept with the y-axis corresponds to σ_{ref} of the reference compound and m is the slope. The absence of sufficiently large set of data for proton chemical shifts prevents the use of the linear regression method for this nucleus. Hence, relative isotropic proton (^1H) chemical shifts were obtained by referencing computed shielding constants against those of the standard tetramethylsilane (TMS) according to $\delta_{iso}(^1H) = \sigma_{ref}(TMS) - \sigma_{iso}$,[35] where σ_{ref}(TMS) was calculated to be 31.15 ppm (cubic supercell with length 20 Å).

2. Detailed Syntheses

2.1 Synthesis of [Rh($Cy_2PCH_2CH_2PCy_2$)(η^6-1,2-$F_2C_6H_4$)][BAr^F_4]

A Schlenk flask was charged with [Rh(COD)$_2$][BAr^F_4] (250 mg, 0.211 mmol) and another filled with $Cy_2PCH_2CH_2Cy_2$ (107 mg, 0.253 mmol). Both solids were dissolved in CH_2Cl_2 (25 ml each) and the phosphine was added to [Rh(COD)$_2$][BAr^F_4] with vigorous stirring to form [Rh($Cy_2PCH_2CH_2PCy_2$)(η^2:η^2-COD)$_2$][BAr^F_4]. The resultant solution was filtered via cannula and to it an excess of pentane was added to triturate the [Rh($Cy_2PCH_2CH_2PCy_2$)(η^2:η^2-COD)$_2$][BAr^F_4] whilst removing impurities. The CH_2Cl_2:Pentane mixture was removed and the solid washed three times more with pentane (3 x 20 ml). The subsequent solid was dried under vacuum before being taken up in a minimum volume of 1,2-$F_2C_6H_4$ (c. 20 ml). The solution was filtered via cannula into a Young's flask before being freeze-pump-thaw degassed three times and the H_2 was added (2 atm). The reaction mixture was allowed to stir for four hours, during which time the solution paled to a yellow colour. After four hours the flask was placed under argon and pentane was added to triturate the desired product as a yellow microcrystalline solid. This was washed with a further three batches of pentane (3 x 20 ml). [Rh($Cy_2PCH_2CH_2PCy_2$)(η^6-1,2-$F_2C_6H_4$)][BAr^F_4] was judged pure by ^{1}H-NMR. Yield = 240 mg, 0.160 mmol, 76 %.

^{1}H NMR (CD_2Cl_2, 500 MHz): 7.72 (s, 8H, ortho-BAr^F_4), 7.56 (s, 4H, para-BAr^F_4), 6.85 (m, diFB), 6.25 (m, 2H, diFB) 6.12 (m, 1H, para-diFB), 2.00 - 1.50 (multiple overlapping aliphatic resonances, 28 H), 1.35 – 0.70 (multiple overlapping aliphatic resonances, 20H).

^{31}P{^{1}H} NMR (CD_2Cl_2, 202 MHz): 98.58 (d, J_{Rh-P} = 199 Hz).

^{19}F{^{1}H} NMR (CD_2Cl_2, 282 MHz): -62.86 (BAr^F_4), -145.55 (d, J_{Rh-F} = 4 Hz).

ESI-MS found (calculated): *m/z* = 639.2631 (639.2562).

Elemental Analysis found (calculated): C 50.99 (51.12), H 4.23 (4.29).

2.2 Synthesis of [Rh($Cy_2PCH_2CH_2PCy_2$)(1,3-η^2:η^2-C_5H_8)][BAr^F_4], [2][BAr^F_4]

An analogous synthesis to that for [Rh($Cy_2PCH_2CH_2PCy_2$)(η^6-1,2-$F_2C_6H_4$)][BAr^F_4] was carried out on the same scale: [Rh(COD)$_2$][BAr^F_4] (250 mg, 0.211 mmol) with $Cy_2PCH_2CH_2Cy_2$ (107 mg, 0.253 mmol). However instead of isolating the yellow solid it was taken up in CH_2Cl_2 (20 ml) and filtered (via cannula) into a Schlenk. The Young's flask was washed with CH_2Cl_2 (3 x 10 ml). The solution was stirred and 1,4-pentadiene (0.1 ml, 2.23 mmol) was added via syringe. The murky yellow solution immediately darkened to a deep burgundy. The solution was allowed to stir for a further 16 hours to ensure all the pentadiene had isomerized to a single product. Following this the solution was triturated with an excess of pentane giving a burgundy solid, the supernatant was subsequently removed and the solid was washed three times with pentane (3 x 20 ml). The solid was dried *in vacuo* and identified by ^{1}H NMR as pure micro-crystalline [Rh($Cy_2PCH_2CH_2PCy_2$)(1,3-η^2:η^2-C_5H_8)][BAr^F_4], **[2][BAr^F_4]**. Yield = 180 mg, 0.124 mmol, 58.6 %. Single crystals suitable for X-ray diffraction can be made by layering a saturated CH_2Cl_2 with hexane and storing at 5 °C for two weeks.

^{1}H NMR (CD_2Cl_2, 500 MHz): 7.72 (s, 8H, ortho-BAr^F_4), 7.57 (s, 4H, para-BAr^F_4), 5.60 (d of d, 1H, J = 4.64 Hz, J = 14.01 Hz, pentadiene-C3), 5.25 (quintet of m, J = 7 Hz, 1H, pentadiene-C2), 4.16 (two

overlapping m, 2H, pentadiene-C4 and C1, H *cis* to H on C2), 2.76 (d, J = 14 Hz, 1H, pentadiene-C1, H *trans* to H on C2), 2.20-1.50 (multiple overlapping aliphatic resonances, 33 H), 1.40 – 0.90 (multiple overlapping aliphatic resonances, 22H).

$^{31}P\{^1H\}$ solution NMR (CD_2Cl_2, 202 MHz): 84.7 (d of d, $J_{Rh\text{-}P}$ = 175 Hz, $J_{P\text{-}P}$ = 23 Hz), 77.1 (d of d, $J_{Rh\text{-}P}$ = 172 Hz, $J_{P\text{-}P}$ = 23 Hz).

$^{13}C\{^1H\}$ solution NMR (CD_2Cl_2, 125.7 MHz): 161.8 (BAr^F_4, ipso-C, q, $J_{B\text{-}C}$ = 51 Hz), 134.9 (BAr^F_4, ortho-C), 125.9 (BAr^F_4, meta-C or CF_3), 123.2 (BAr^F_4, meta-C or CF_3), 117.3 (BAr^F_4, para-C), 105.3 (pentadiene, C3), 96.7 (pentadiene, C2), 92.0 (pentadiene, C4), 61.3 (pentadiene, C1), 21-40 (multiple aliphatic resonances), 20.2 (pentadiene-CH_3, C5).

$^{31}P\{^1H\}$ SSNMR (161.9 MHz): 86.5 (d, $J_{Rh\text{-}P}$ = 146 Hz), 82.1 (d, $J_{Rh\text{-}P}$ = 140 Hz), 77.8 (d, $J_{Rh\text{-}P}$ = 140 Hz), 74.1 (br).

$^{13}C\{^1H\}$ SS NMR (100.6 MHz): 164.3 (BAr^F_4, ipso-C), 135.0 (BAr^F_4), 134.0 (BAr^F_4), 129.6 (BAr^F_4), 124.2 (BAr^F_4), 117.2 (BAr^F_4), 115.6 (BAr^F_4), 104.0 (Pentadiene), 102.9 (Pentadiene), 96.2 (Pentadiene), 94.1 (Pentadiene), 91.4 (Pentadiene), 87.9 (Pentadiene), 60.2 (Pentadiene), 16-40 (multiple overlapping aliphatic resonances).

ESI-MS found (calculated): *m/z* = 593.2912 (593.2907).

Elemental Analysis found (calculated): C 51.76 (51.91), H 4.55 (4.71).

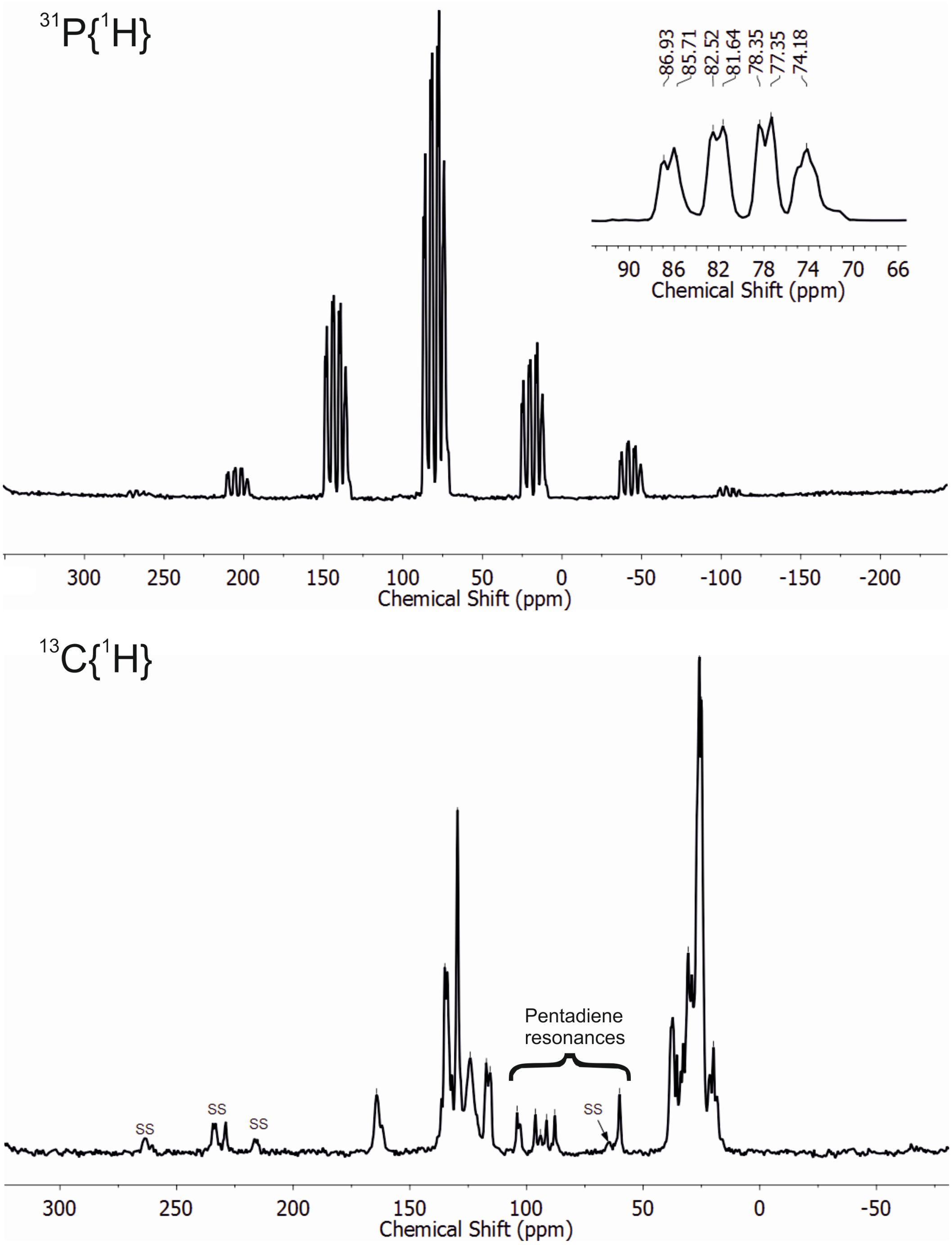

Figure S1: The $^{31}P\{^1H\}$ (top) and $^{13}C\{^1H\}$ (bottom) SSNMR spectra of **[2][BAr$^F_4$]**. The insert in the ^{31}P spectrum is of the central resonance (the others being spinning side bands). The pentadiene alkene resonances and resonances attributed to spinning sidebands (SS) are highlighted in the ^{13}C spectrum.

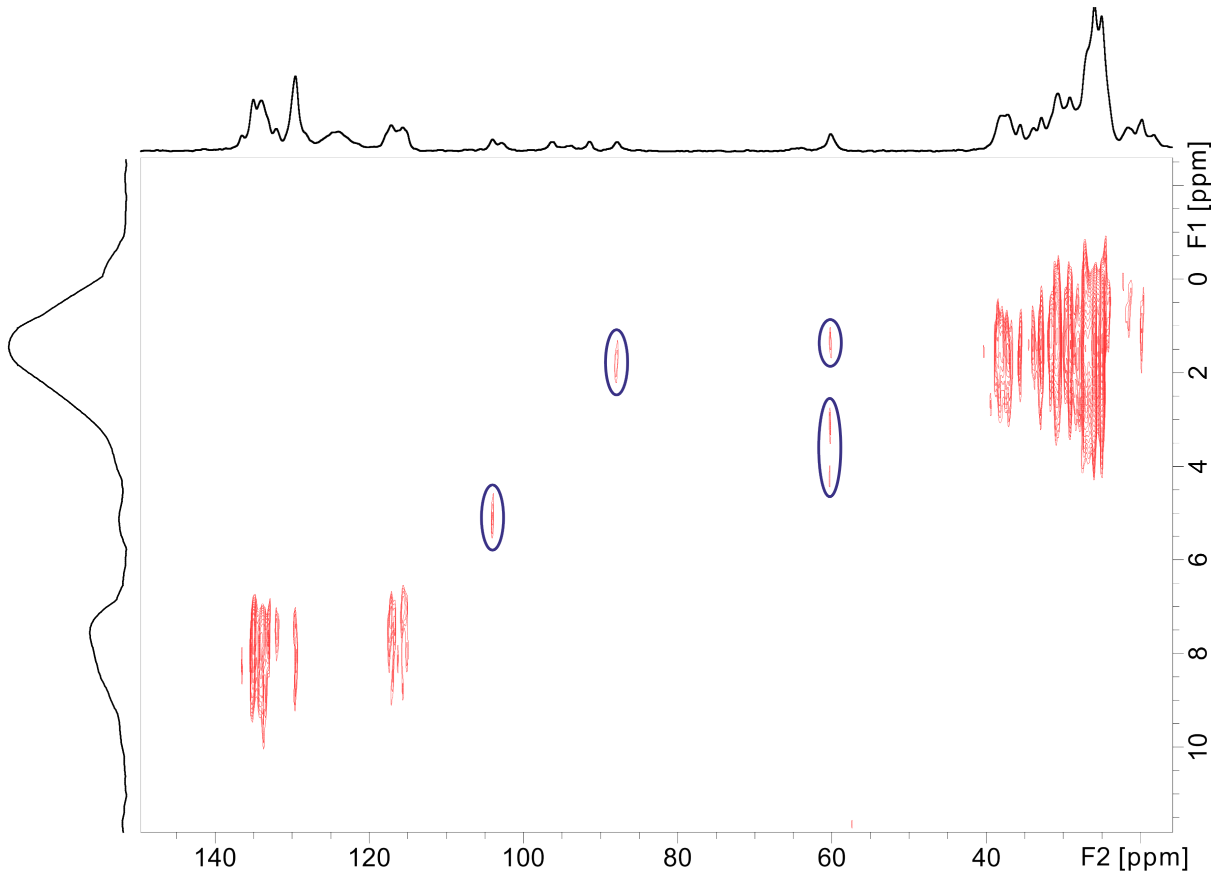

Figure S2: The FSLG-HETCOR SSNMR of **[2][BAr$^F_4$]**. Highlighted resonances are attributed to the pentadiene fragment. Spectrum collected at 298 K.

3. Hydrogenation of [2][BAr$^F_4$]

3.1 Solution phase hydrogenation

A sample of **[2][BAr$^F_4$]** (c. 10 mg) was loaded into a high pressure NMR tube in a glovebox. This was subsequently dissolved in CD_2Cl_2, and then freeze-pump-thaw-degassed three times and to the frozen tube H_2 was added (2 atm when at room temperature). The burgundy solution quickly turned yellow and NMR experiments showed primarily the formation of mainly the previously characterised [Rh($Cy_2PCH_2CH_2PCy_2$){(η^6-C_6H_3(3,5-CF_3)$_2$)BAr$^F_3$}] - demonstrated by ^{31}P{^{1}H} resonance at δ 91.5 ($J_{P\text{-}Rh}$ = 200 Hz; figure S3 bottom) and ^{1}H resonances characteristic of bound BAr$^F_4$ at δ 7.65, 7.51, 7.24 and 7.08 (3:6:1:2 integral ratio, figure S3 top).[12] Along with this compound other unidentified species are formed, presumed to be C-D or C-Cl activation products (as had been previously observed).[12]

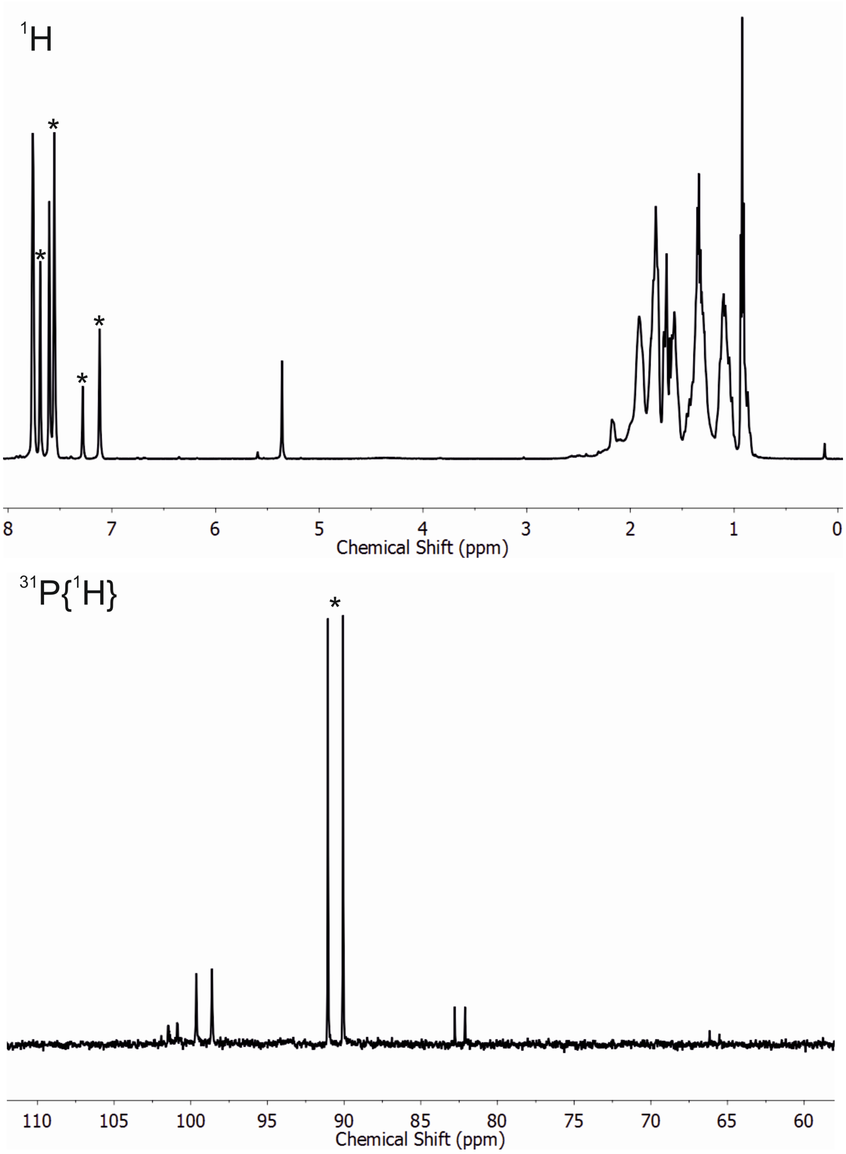

Figure S3: The ^{1}H and ^{31}P{^{1}H} solution NMR spectrum of the hydrogenation of **[2][BAr$^F_4$]** in the solution phase. The main product (indicated by *) is that of the known BAr$^F_4$ complex

$[Rh(Cy_2PCH_2CH_2PCy_2)\{\eta^6\text{-}C_6H_3(3,5\text{-}CF_3)BAr^F{}_3\}]$, **[4]**.[12] Other products are unassigned. Spectrum collected at 298 K.

3.2 Solid state hydrogenation: synthesis of [3][BAr$^F{}_4$]

Addition of hydrogen (2 atm) to a crystalline sample of **[2][BAr$^F{}_4$]** for two minutes afforded complete conversion to **[3][BAr$^F{}_4$]**. Quick transfer of a single crystal (< 10 mins) to a diffractometer allowed for the measurement of a single-crystal X-ray structure. Crystals retained crystallinity indefinitely at 150 K, however if kept at room temperature data quality would decrease, with any sample left for 30 mins not having sufficient quality data to solve (Figure S13).

For SSNMR measurements crystalline samples were ground up in a glove box using the back of a spatula then pre-packed into a rotor. The rotor was loaded into a custom piece of glassware, and sat on top of a cap (however the rotor was not sealed). H_2 (2 atm) was admitted to the glassware and it was kept open for 210 seconds. The glassware was then sealed and cooled to 195 K to halt the reaction and forestall decomposition. The vessel was then pumped onto an argon Schlenk line, and under a flush of argon the rotor was pushed onto the cap, sealing the rotor, whilst allowing it to remain as cold as possible. The glassware was kept at 195 K whilst transported to the pre-cooled spectrometer (223 K).

In order to collect spectra at 158 K (Figures S5, S6, S7 and S9) a sample was prepared as above and loaded into the spectrometer at 223 K. Experimentally it was found that if the spectrometer was cooled any further the sample would not spin. A short $^{31}P\{^1H\}$ SSNMR scan was run in order to check the quality of the sample (Figure S9), before the spectrometer was cooled to 158 K. Once the temperature stabilised FSLG-HETCOR, $^{13}C\{^1H\}$ and $^{31}P\{^1H\}$ SSNMR spectra were collected (Figures S5, S6, S7 and S9). The sample was then incrementally warmed back to 223 K (Figure S7), before being warmed to room temperature (Figure S9).

$^{31}P\{^1H\}$ SSNMR (223 K, 161.9 MHz): 107.6 (d, $J_{Rh\text{-}P}$ = 238 Hz), 104.1 (d, $J_{Rh\text{-}P}$ = 232 Hz).

$^{13}C\{^1H\}$ SSNMR (223 K, 100.6 MHz): 163.0 (BAr$^F{}_4$, ipso-C), 133.7 (BAr$^F{}_4$), 129.1 (BAr$^F{}_4$), 124.0 (BAr$^F{}_4$), 117.7 (BAr$^F{}_4$), 115.4 (BAr$^F{}_4$), 12-40 (multiple overlapping aliphatic resonances).

$^{31}P\{^1H\}$ SSNMR (158 K, 161.9 MHz): 107.2 (d, $J_{Rh\text{-}P}$ = 198 Hz), 104.1 (d, $J_{Rh\text{-}P}$ = 198 Hz).

$^{13}C\{^1H\}$ SSNMR (158 K, 100.6 MHz): 163.0 (BAr$^F{}_4$, ipso-C), 133.7 (BAr$^F{}_4$), 128.7 (BAr$^F{}_4$), 123.6 (BAr$^F{}_4$), 116.4 (BAr$^F{}_4$), 7-40 (multiple overlapping aliphatic resonances).

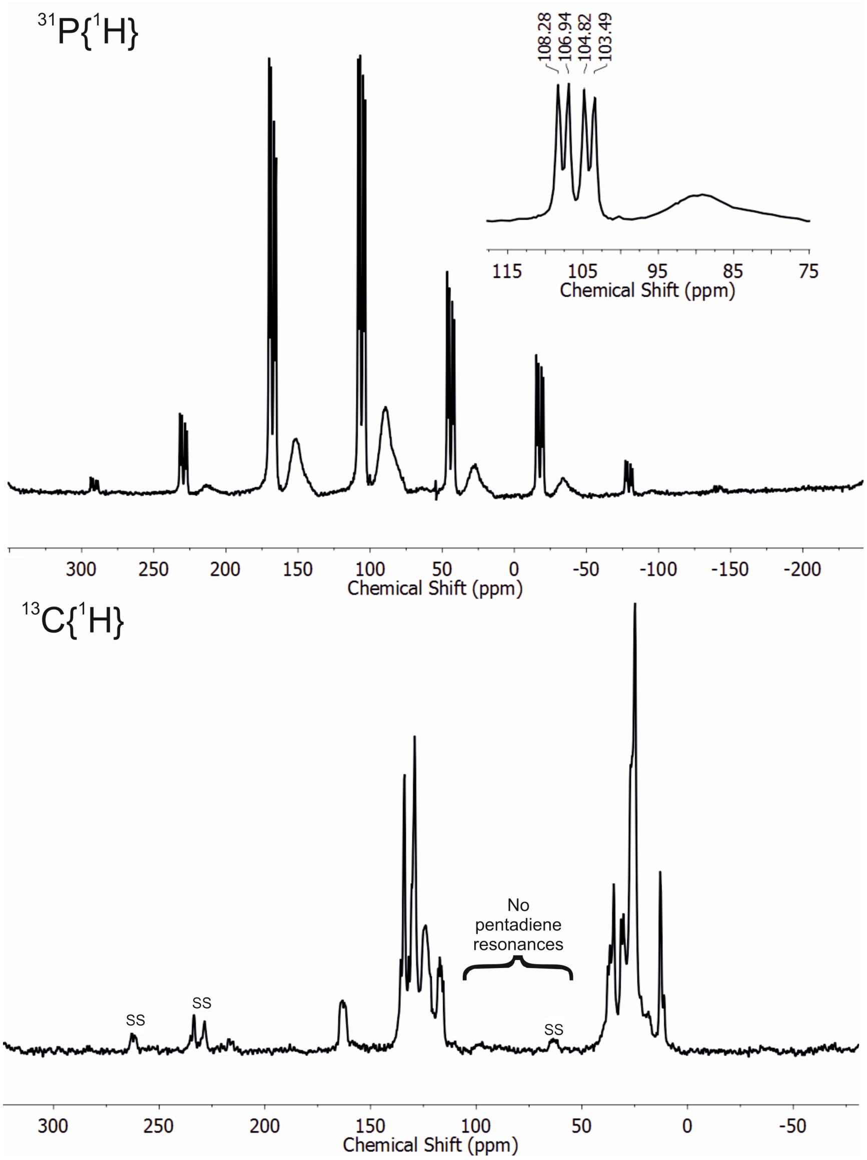

Figure S4: The $^{31}P\{^{1}H\}$ (top) and $^{13}C\{^{1}H\}$ (bottom) SSNMR spectra of **[3][BAr^{F}$_4$]**. The insert in the ^{31}P spectrum is of the central resonance (the broad hump is the decomposition product, [Rh($Cy_2PCH_2CH_2PCy_2$){η^6-C_6H_3(3,5-CF_3)BAr^{F}$_3$}], **[4]**[12] and the other sharp resonances are spinning sidebands). The absence of pentadiene alkene resonances is highlighted in the ^{13}C spectrum. Spectra were collected at 223 K.

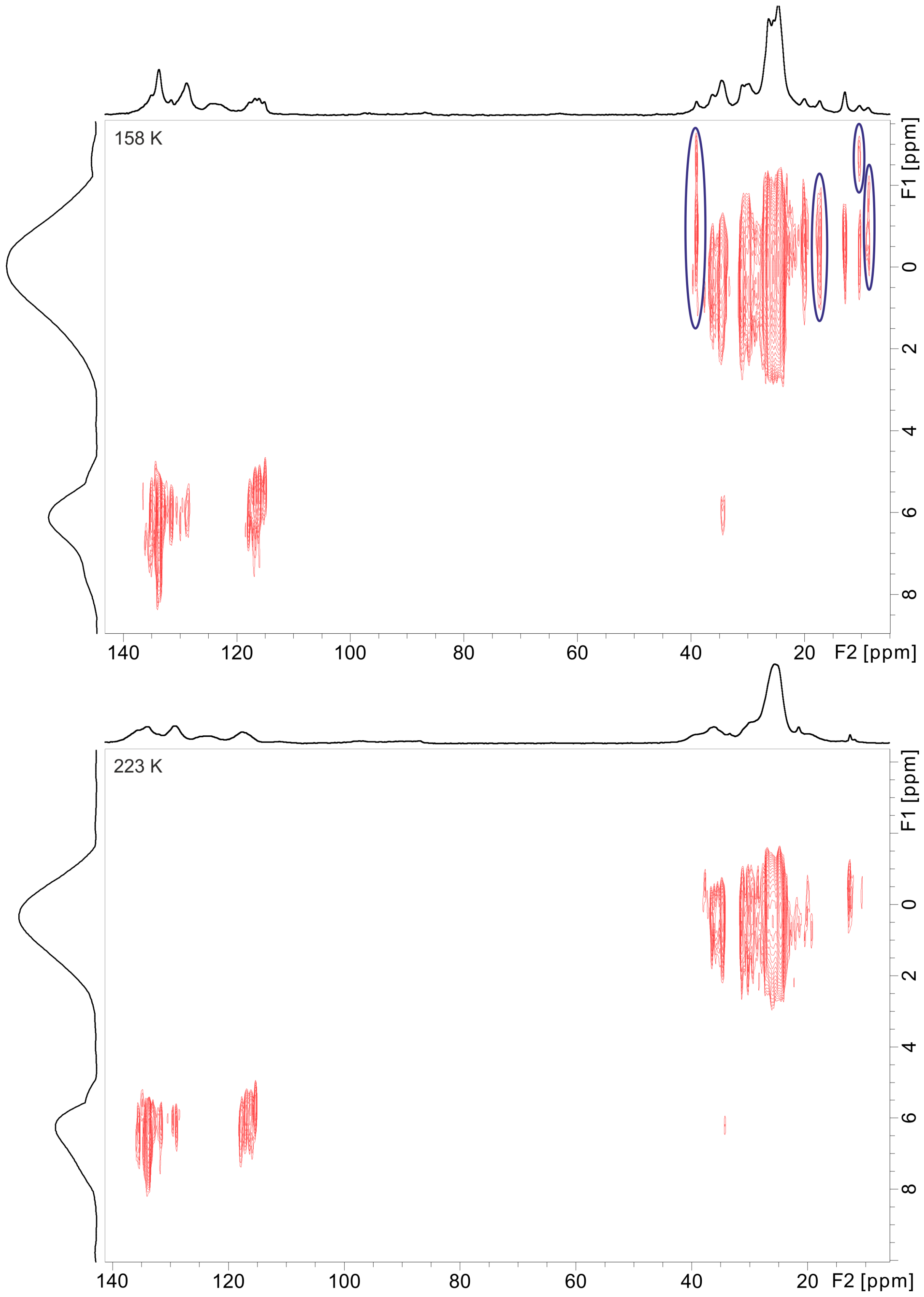

Figure S5: The FSLG-HETCOR SSNMR spectra of **[3][BAr$^F{}_4$]** at 158 K (top) and then warmed to 223 K (bottom). The highlighted resonances in the top spectrum are absent in the bottom spectrum.

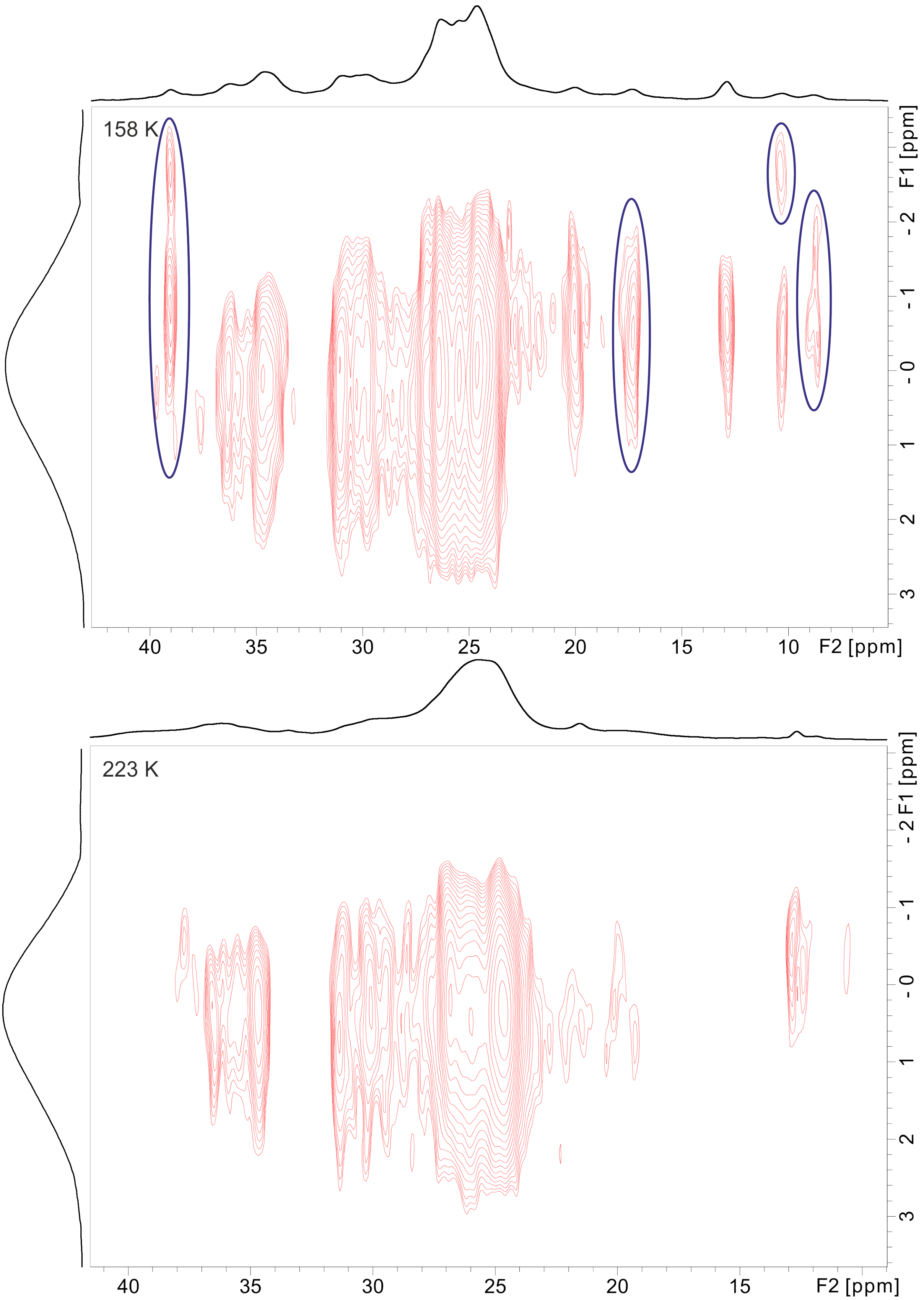

Figure S6: The FSLG-HETCOR SSNMR spectra of **[3][BAr$^F{}_4$]** at 158 K (top) and then warmed to 223 K (bottom). The highlighted resonances in the top spectrum are absent in the bottom spectrum.

Table S1: ^{13}C:^{1}H correlations from the FSLG-HETCOR SSNMR measurements of **[3][BAr$^{F}_{4}$]** at various temperatures. Measurements were undertaken at 158 K, 223 K and then subsequently of the decomposition product **[4]**[12] (having been stored at room temperature for a week).

**158 K**			**223 K**			**298 K** (after 1 week – i.e. decomposition)	
^{13}**C**	^{1}**H**		^{13}**C**	^{1}**H**		^{13}**C**	^{1}**H**
135.0	6.09		135.5	6.36		135.0	6.78
133.6	6.07		133.7	6.28		134.3	6.90
131.5	5.99		131.6	6.19		133.5	6.38
128.9	6.04		129.0	6.09			
117.8	5.90		117.7	6.13		116.8	6.43
116.7	5.73		116.9	6.01			
116.0	5.80		116.2	5.76			
115.1	5.46		115.4	5.76			
39.0	-2.69						
39.0	-0.90		37.6	-0.53		35.8	0.78
36.3	0.07		36.5	0.04			
34.6	0.14		34.7	0.13			
31.0	0.12		31.3	0.55			
30.2	0.09		30.1	0.23			
26.3	0.19		26.0	0.42		25.6	0.33
24.6	-0.13		24.7	0.31			
20.1	-0.53		19.9	-0.11			
17.4	-0.57						
12.9	-0.62		12.8	-0.39			
10.3	-2.74		10.7	-0.15			
10.3	-0.38						
8.7	-1.96						
8.7	-0.60						

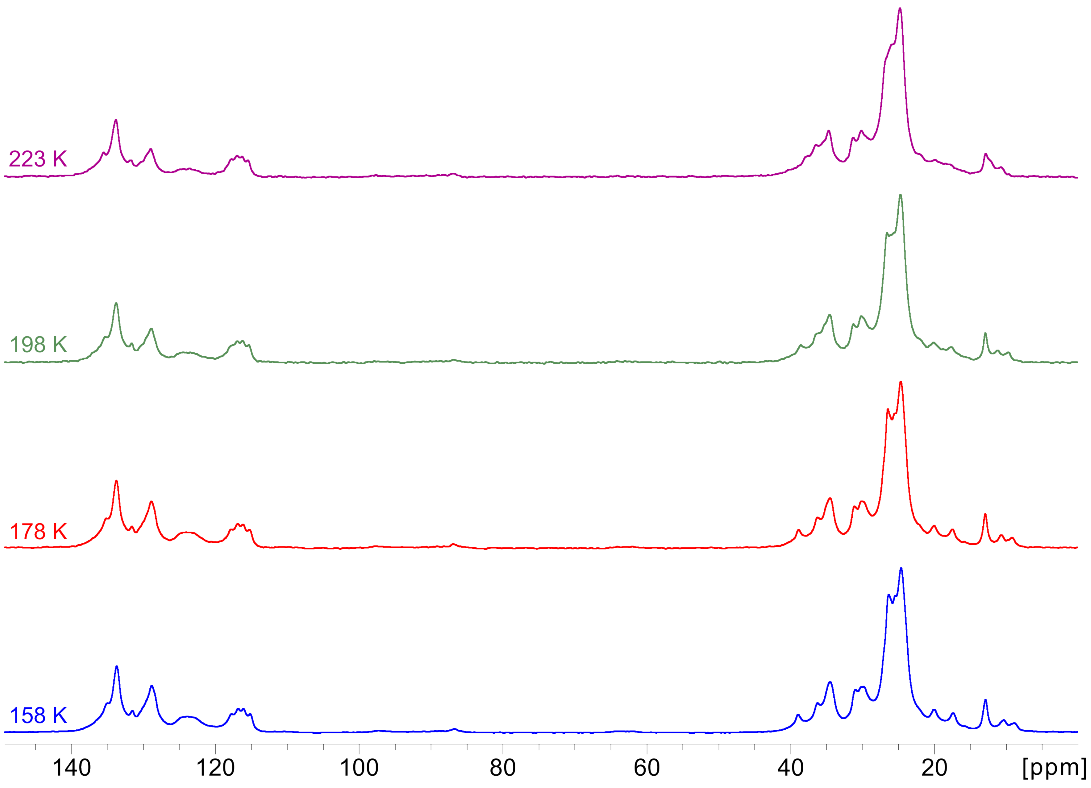

Figure S7: Variable temperature $^{13}C\{^1H\}$ SSNMR spectra of **[3][BAr$^F_4$]** upon warming from 158 K to 223 K demonstrating the broadening of the ^{13}C signals.

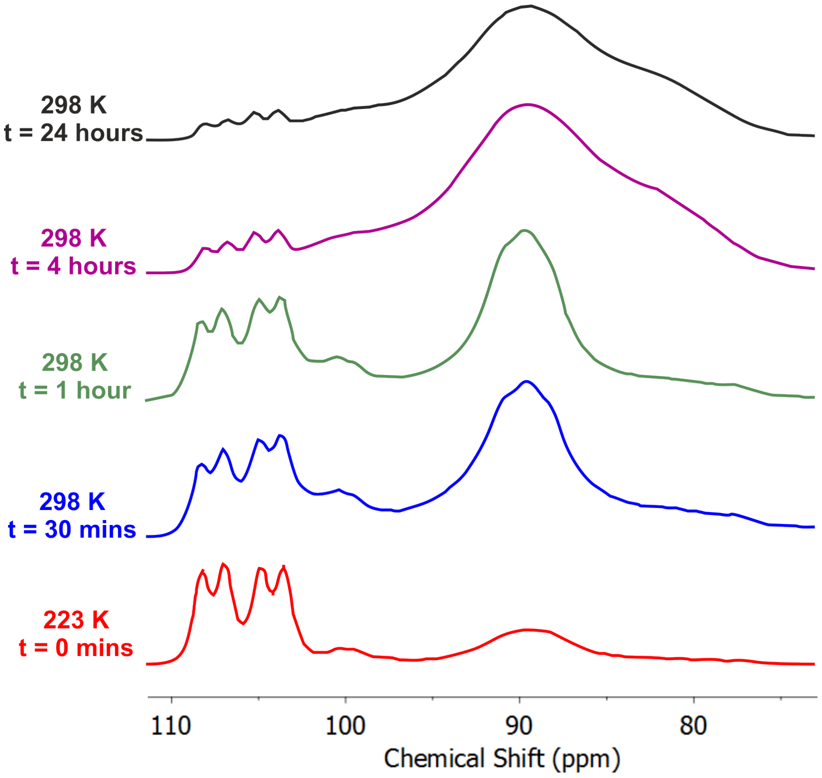

Figure S8: Sequential $^{31}P\{^1H\}$ SSNMR spectra of **[3][BAr$^F_4$]**. The broad hump is the decomposition product, assigned to [Rh($Cy_2PCH_2CH_2PCy_2$){(η^6-C_6H_3(3,5-CF_3)$_2$)BAr$^F_3$}], **[4]**.[12] The bottom spectrum is the initial spectrum (collected at 223 K).

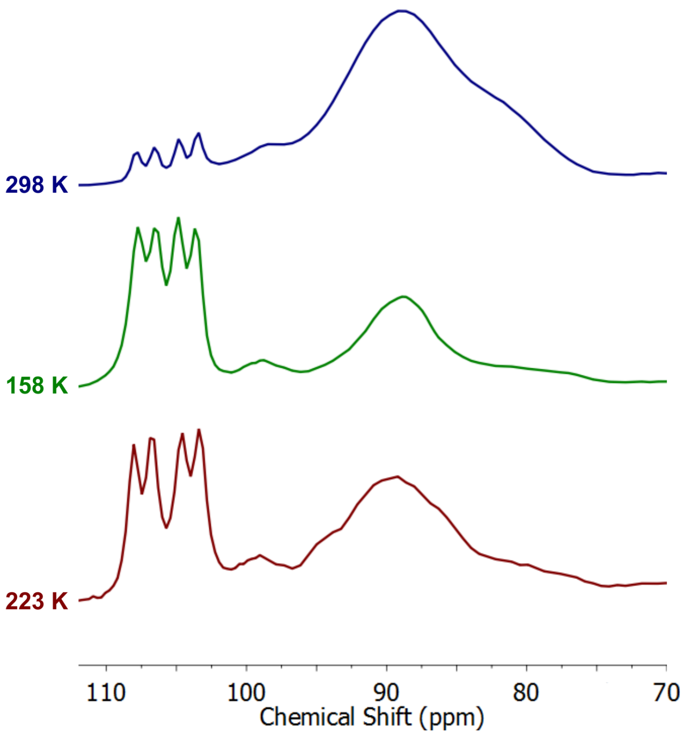

Figure S9: Monitoring the effect of cooling a sample of **[3][BArF4]** by $^{31}P\{^{1}H\}$ SSNMR. The broad hump is the decomposition product, $[Rh(Cy_2PCH_2CH_2PCy_2)\{(\eta^6\text{-}C_6H_3(3,5\text{-}CF_3)_2)BAr^F{}_3\}]$, **[4]**.[12] The bottom spectrum is the initial spectrum (collected at 223 K), the sample is then cooled to 158 K over a period of approximately 1 hour (middle spectrum). The top spectrum is of the same sample warmed to room temperature over a period of approximately 4 hours.

4. Dissolution of [3][BAr$^F_4$] at room temperature

If the hydrogenation products are dissolved in CD_2Cl_2 the primary product formed is the bound BAr$^F_4$ species [Rh($Cy_2PCH_2CH_2PCy_2$){(η^6-C_6H_3(3,5-CF_3)$_2$)BAr$^F_3$}], **[4]** (figure S10) and concomitant unidentified C-D or C-Cl activation products.[12] Similar resonances were observed when the equivalent experiment was conducted on the previously reported [Rh($Cy_2PCH_2CH_2PCy_2$)(η^2:η^2-C_7H_{12})][BAr$^F_4$] **[1b][BAr$^F_4$]**.[12]

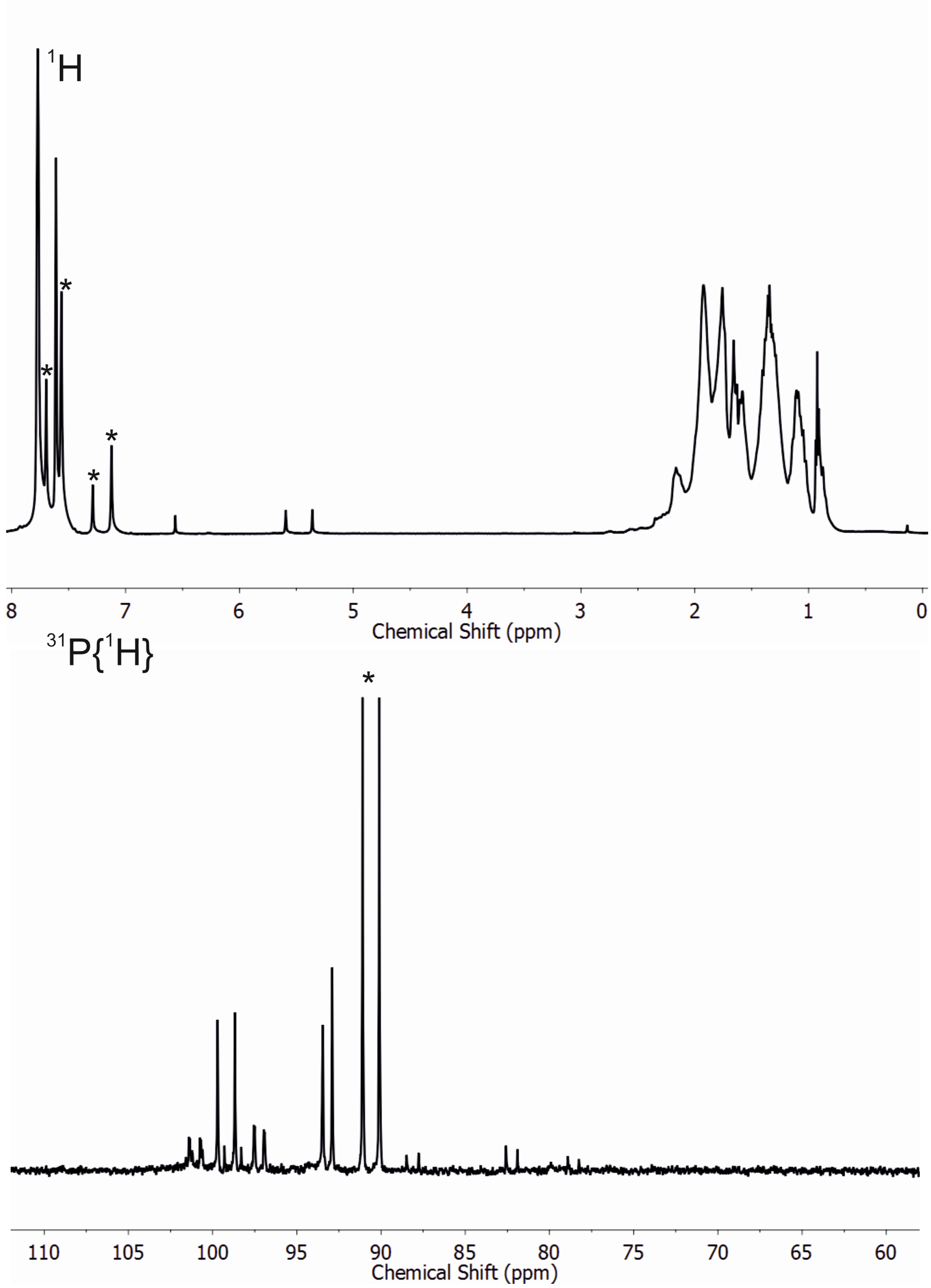

Figure S10: The ^{1}H solution NMR spectrum (top) and ^{31}P{^{1}H} solution NMR spectrum (bottom) of [3][BAr$^F_4$] dissolved in CD_2Cl_2. The primary product (*) is the bound BAr$^F_4$ species [Rh($Cy_2PCH_2CH_2PCy_2$){(η^6-C_6H_3(3,5-CF_3)$_2$)BAr$^F_3$}], **[4]**.[12] The spectra were recorded at 298 K.

5. Dissolution of [3][BAr$^F_4$] at low temperature

CD_2Cl_2 was condensed onto the hydrogenation products at 77 K in an NMR tube. The frozen liquid was thawed and the crystalline sample was dissolved before refreezing. The spectrometer was pre-cooled to 183 K. The sample was then thawed and immediately placed into the spectrometer. After the temperature was allowed to stabilize (approximately two minutes) spectra were collected (Figure S11). At this temperature no [Rh($Cy_2PCH_2CH_2PCy_2$){(η^6-C_6H_3(3,5-CF_3)$_2$)BAr$^F_3$}], **[4]** was observed. It is posited that the product is that of a CD_2Cl_2 complex that could not be isolated (^{31}P{^{1}H} δ 98.4 $J_{Rh\text{-}P}$ = 203 Hz). This hypothesis is reinforced as analogous experiments undertaken upon the previously synthesized [Rh($Cy_2PCH_2CH_2PCy_2$)(η^2:η^2-C_7H_{12})][BAr$^F_4$] **[1b][BAr$^F_4$]** gave similar spectra. The sample was then warmed to 233 K and [Rh($Cy_2PCH_2CH_2PCy_2$){(η^6-C_6H_3(3,5-CF_3)$_2$)BAr$^F_3$}], **[4]** was observed to grow in (Figure S12).

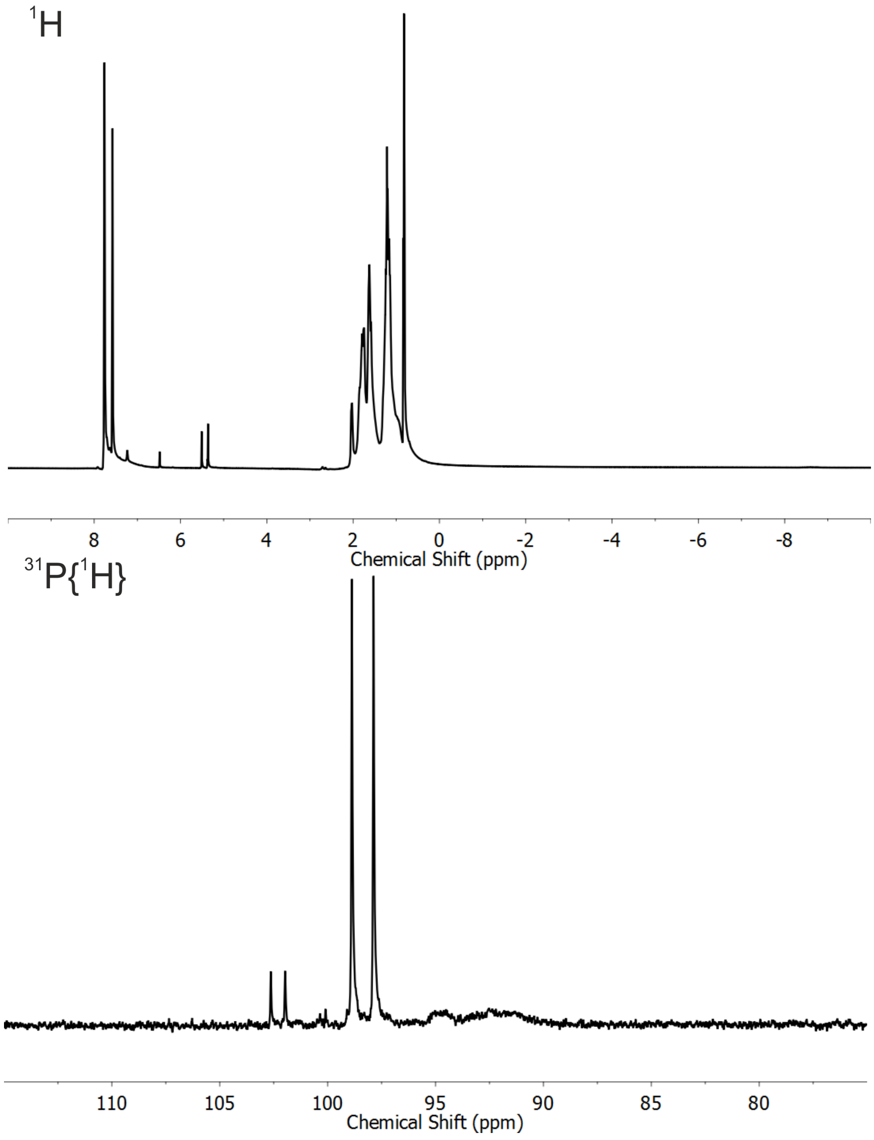

Figure S11: The ^{1}H (top) and ^{31}P{^{1}H} (bottom) solution NMR spectra of **[3][BAr$^F_4$]** dissolved in CD_2Cl_2 at 183 K. The primary product is the same as that observed when the analogous experiment is carried out on [Rh($Cy_2PCH_2CH_2PCy_2$)(η^2:η^2-C_7H_{12})][BAr$^F_4$] **[1b][BAr$^F_4$]**. [12] The spectra were recorded at 183 K.

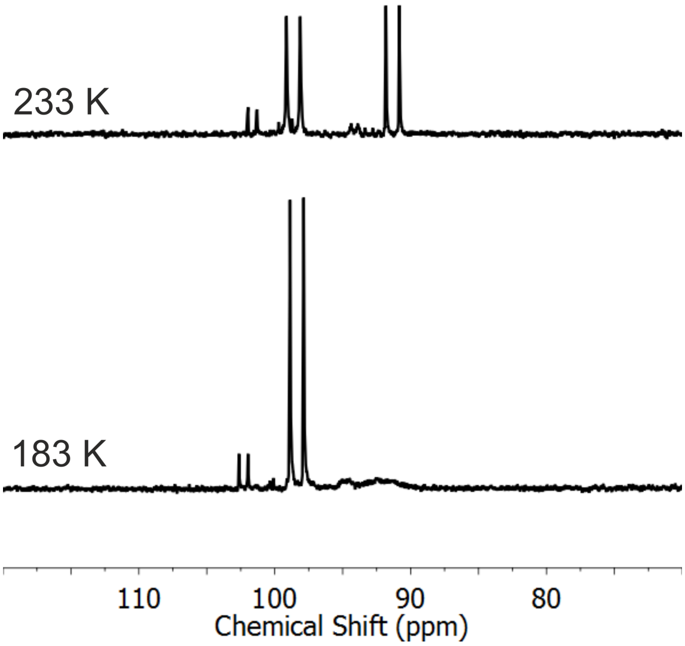

Figure S12: The $^{31}P\{^{1}H\}$ (bottom) solution NMR spectra of **[3][BAr$^{F}_{4}$]** dissolved in CD_2Cl_2 at 183 K and subsequently warmed to 233 K. The resonance that grows in is corresponds to the previously synthesized [Rh($Cy_2PCH_2CH_2PCy_2$){(η^6-C_6H_3(3,5-CF_3)$_2$)BAr$^F_3$}], **[4]**.[12]

6. Crystallographic information

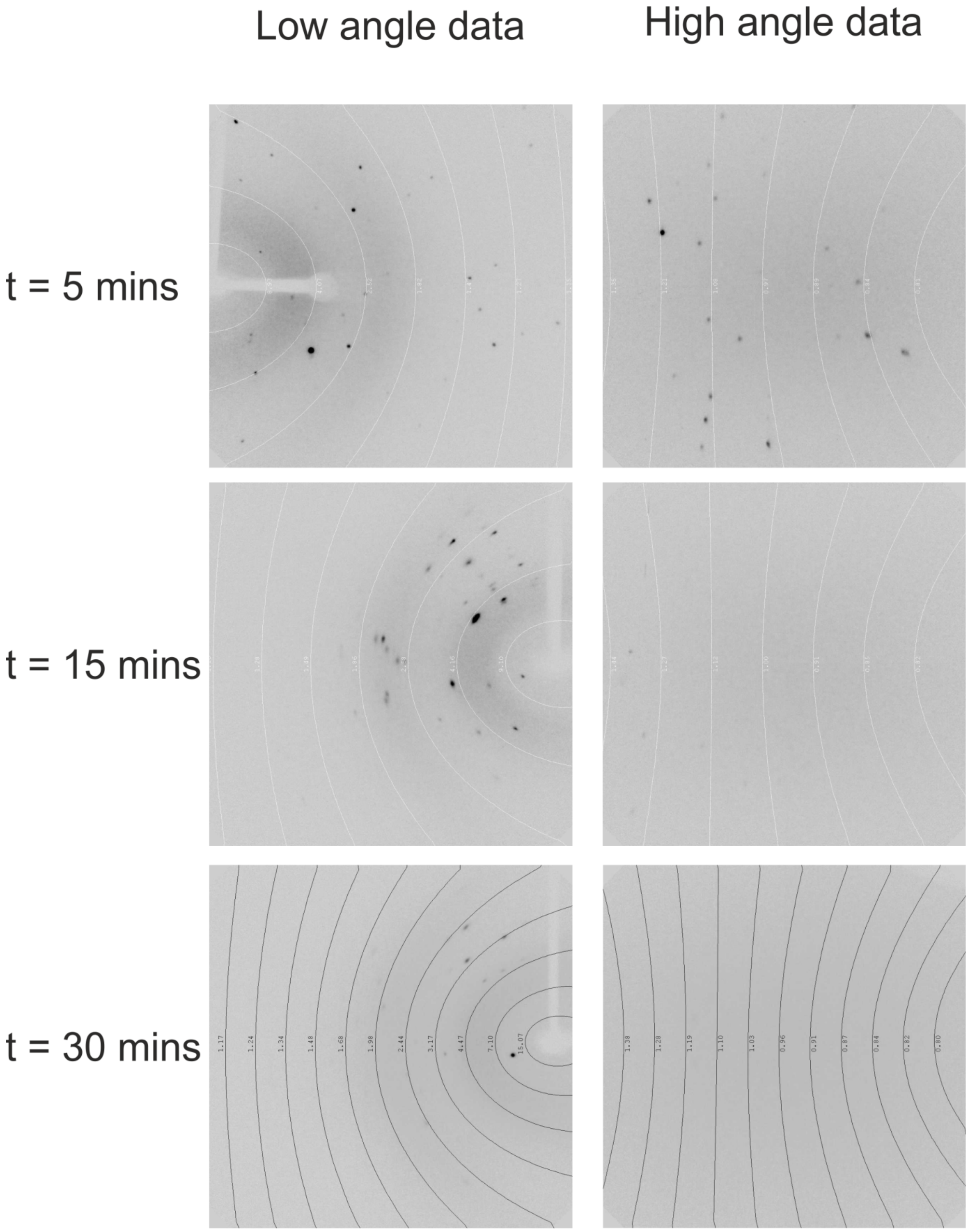

Figure S13: Low angle and high angle crystal frames of hydrogenation products. The samples were hydrogenated in high pressure NMR tubes and left in the tubes at 298 K for the stated time period before quick transfer to the diffractometer at 150K. Low angle frames were exposed at 4 s exposure and high angle frames were at 16 s exposure. Crystals were of comparable size and quality (approx. 0.1 x 0.1 x 0.1 mm). All data collected at 150 K.

Table S2: Selected single crystal X-ray diffraction experimental parameters.

	**[2][BAr$^F_4$]**	**[3][BAr$^F_4$]**
Chemical formula	$C_{31}H_{55}P_2Rh$; $C_{32}H_{12}BF_{24}$	$C_{31}H_{60}P_2Rh$; $BC_{32}H_{12}F_{24}$
M_r	1455.84	1460.88
a, *b*, *c* (Å)	12.8345 (3)<br>13.2654 (3)<br>20.0408 (4)	13.0259 (2)<br>13.5044 (3)<br>19.1472 (3)
α, β, γ (°)	100.5894 (16)<br>97.7450 (17)<br>100.7599 (17)	99.8660 (16)<br>93.5734 (15)<br>99.4334 (16)
V (Å^3)	3244.07 (13)	3259.25 (11)
μ (mm^{-1})	3.56	3.55
Crystal size (mm)	0.15 × 0.10 × 0.05	0.10 × 0.10 × 0.05
T_{min}, T_{max}	0.40, 0.84	0.40, 0.84
No. of measured, independent and observed [$I > 2.0\sigma(I)$] reflections	34702, 13443, 12330	59080, 13127, 10771
R_{int}	0.024	0.068
(sin θ/λ)$_{max}$ (Å^{-1})	0.630	0.627
$R[F^2 > 2\sigma(F^2)]$, $wR(F^2)$, S	0.072, 0.177, 0.86	0.056, 0.129, 0.97
No. of reflections	13390	13071
No. of parameters	1081	1036
No. of restraints	2278	0
CCDC number	1440135	1440134

7. Optimised geometries

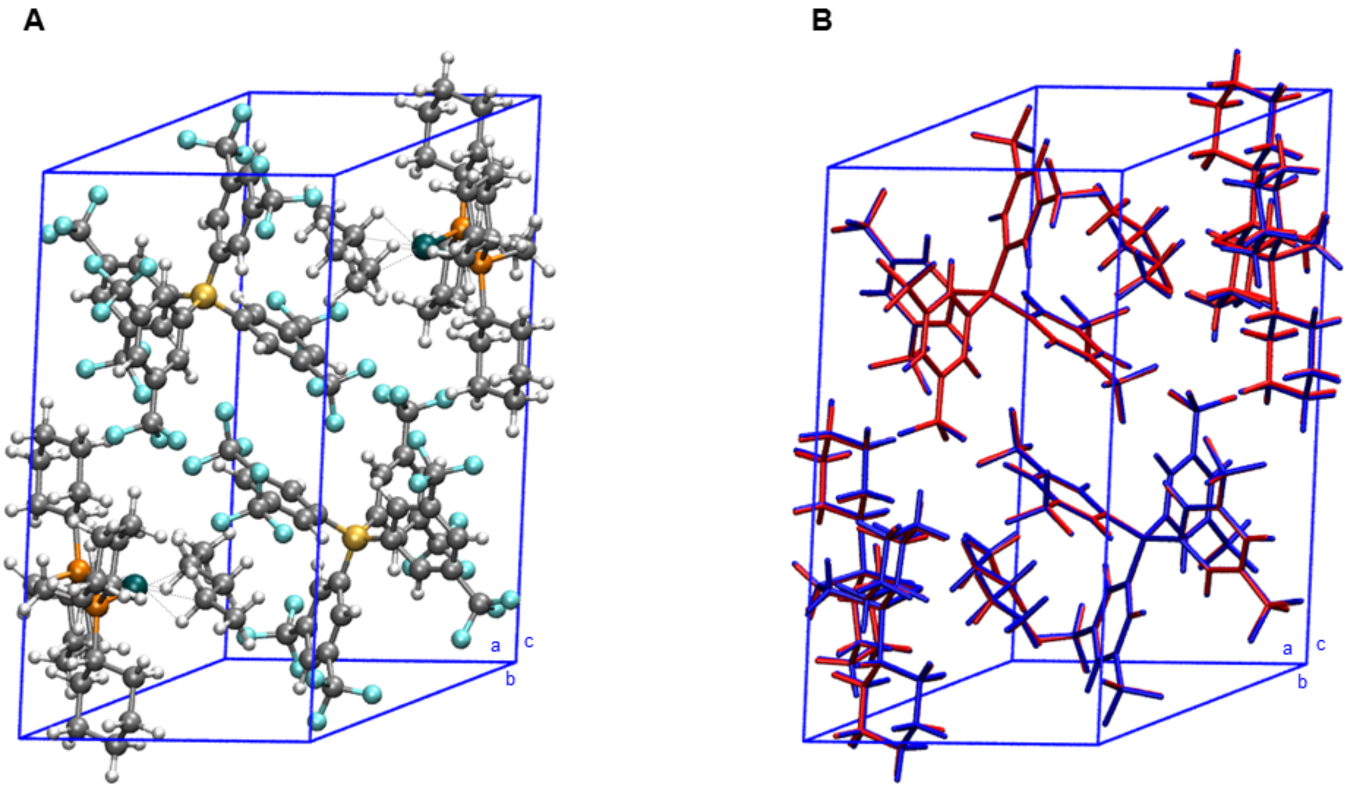

Figure S14: (**A**) DFT-optimized geometry of **[3][BAr$^{F}_{4}$]** showing the unit cell. (**B**) Overlay of the crystal structure of **[3][BAr$^{F}_{4}$]** (red) and the optimized structure (blue).

Table S3: Selected optimized bond distances (Å) and angles (°) for **[3][BAr$^F_4$]** and isolated cation **[3]$^+$**.

Parameter	X-ray	PBE-D3			PBE	
		[3]$^+$	[3][BAr$^F_4$]	[3][BAr$^F_4$] (222)	[3]$^+$	[3][BAr$^F_4$]
Rh1–P1	2.197(10)	2.208	2.207	2.207	2.216	2.206
Rh1–P2	2.196(11)	2.203	2.207	2.207	2.211	2.207
Rh1–C2	2.514(4)	2.517	2.509	2.505	2.560	2.506
Rh1–C4	2.522(5)	2.535	2.534	2.537	2.577	2.525
C1–C2	1.519(7)	1.532	1.531	1.531	1.534	1.530
C2–C3	1.534(7)	1.533	1.533	1.533	1.533	1.532
C3–C4	1.533(7)	1.535	1.532	1.533	1.534	1.532
C4–C5	1.537(7)	1.533	1.533	1.533	1.534	1.532
C2–H21	0.87(5)	1.139	1.137	1.136	1.139	1.135
C2–H22	1.07(5)	1.112	1.114	1.115	1.109	1.115
C4–H41	0.83(5)	1.138	1.141	1.141	1.138	1.141
C4–H42	1.02(5)	1.112	1.109	1.109	1.109	1.109
Rh1–H21	2.24(5)	2.005	2.022	2.029	2.012	2.036
Rh1–H41	2.24(5)	2.034	1.999	1.998	2.037	1.109
Rh1-B1	7.743	-	7.699	7.696	-	7.725
C2–C3–C4	110.4(4)	109.8	109.7	109.7	110.5	109.6
C1–C2–C3	113.8(4)	116.0	115.7	115.7	115.3	115.9
C3–C4–C5	114.3(4)	115.5	115.6	115.6	114.9	115.8
Rh1–P1–P2 / CtoC5	9.5	7.1	9.7	9.5	9.6	8.8

8. AIM analysis

Table S4: Calculated QTAIM parameters (a.u.) for selected bond critical points in **[3][BAr$^F_4$]** and **[1b][BAr$^F_4$]**. Parameters are defined as follows: $\rho(r)$ electron density, $\nabla^2\rho(r)$ Laplacian of electron density, ε ellipticity, V(r) potential energy density, G(r) gradient kinetic energy, H(r) = V(r) + G(r) local energy density.

Complex	BCP	$\rho(r)$	$\nabla^2\rho(r)$	ε	V(r)	G(r)	H(r)
**[3][BAr$^F_4$]**	Rh···H21/H41	0.047 / 0.047	0.172 / 0.174	0.987 / 0.730	-0.052 / -0.054	0.048 / 0.049	–0.004 / –0.005
	C2–H21/ C4–H41	0.247 / 0.243	–0.712 / –0.685	0.025 / 0.026	–0.274 / –0.268	0.048 / 0.048	–0.226 / –0.220
	C2–H22/ C4–H42	0.263 / 0.267	–0.832 / –0.865	0.015 / 0.011	–0.300 / –0.307	0.046 / 0.045	–0.254 / –0.262
	Rh–P1/P2	0.119 / 0.119	0.073 / 0.072	0.013 / 0.025	–0.135 / –0.135	0.076 / 0.076	–0.059 / –0.059
**[1b][BAr$^F_4$]**	Rh···H11/H21	0.058 / 0.060	0.240 / 0.245	1.249 / 1.195	–0.075 / –0.077	0.067 / 0.069	–0.008 / –0.008
	C1–H11/ C2–H21	0.234 / 0.233	–0.621 / –0.615	0.016 / 0.014	–0.252 / –0.251	0.049 / 0.049	–0.203 / –0.202
	C1–H12/ C2–H22	0.278 / 0.277	–0.954 / –0.950	0.002 / 0.002	–0.323 / –0.322	0.042 / 0.042	–0.281 / –0.280
	Rh–P1/P2	0.118 / 0.118	0.078 / 0.078	0.003 / 0.013	–0.135 / –0.134	0.077 / 0.076	–0.058 / –0.058

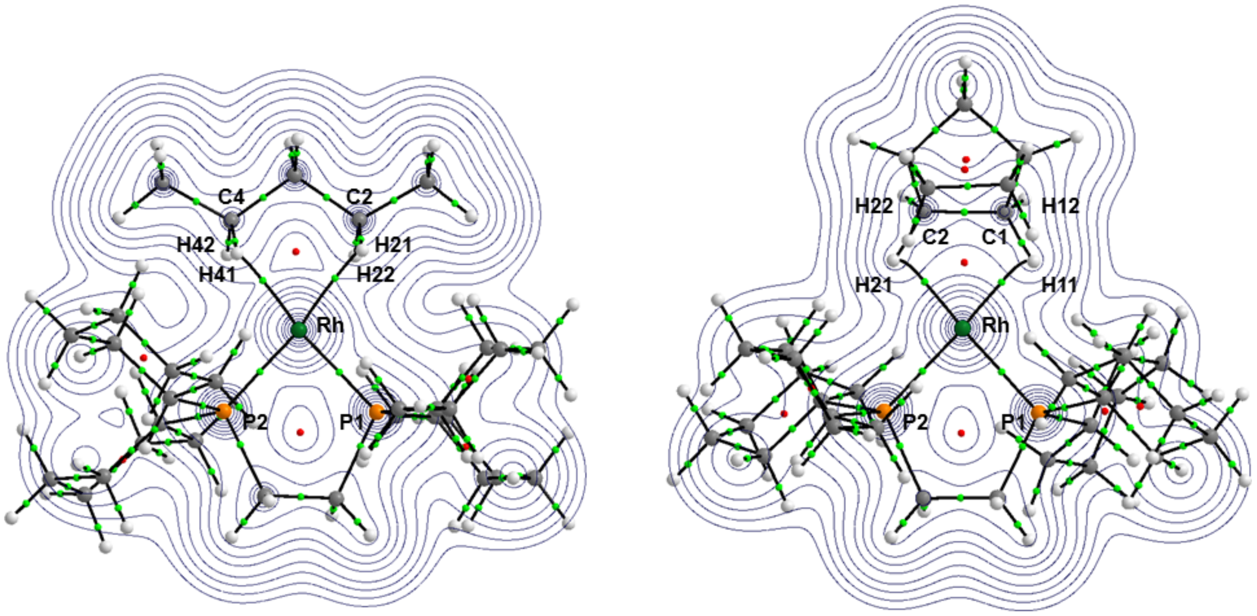

BCP	ρ(r)	∇²ρ(r)	H(r)
**Rh···H21**	0.047	0.172	–0.004
**C2–H21**	0.247	–0.712	–0.226
**C2–H22**	0.263	–0.832	–0.254

BCP	ρ(r)	∇²ρ(r)	H(r)
**Rh···H11**	0.058	0.240	–0.008
**C1–H11**	0.234	–0.621	–0.203
**C1–H12**	0.278	–0.954	–0.281

Figure S15: Contour plots of the electron density (BP86/SDD/6-31G**) of the cationic portions in **[3][BAr$^F_4$]** and **[1b][BAr$^F_4$]** in the {Rh P1 P2} plane, with projected stationary points and bond paths. Bond critical points (BCP) are shown in green and ring critical points (RCP) in red. Calculated QTAIM parameters (a.u.) for selected BCPs are shown ($\rho(r)$ electron density; $\nabla^2\rho(r)$ Laplacian of electron density; H(r) = local energy density).

9. NBO analysis

Table S5: Interaction energies from second-order perturbation theory between key NBOs within the cationic units of **[3][BAr$^F_4$]** and **[1b][BAr$^F_4$]**.

Complex	Donor/acceptor orbitals	$\Delta E^{(2)}$ / kcal mol^{-1}
**[1b][BAr$^F_4$]**	$\sigma_{CH} \rightarrow \sigma^*_{RhP}$ (*trans*)	20.61 / 19.76
	$\sigma_{CH} \rightarrow \sigma^*_{RhP}$ (*cis*)	<0.05 / <0.05
	$LP_{Rh} \rightarrow \sigma^*_{CH}$	3.92 / 3.57
	σ_{RhP} (*cis*) $\rightarrow \sigma^*_{CH}$	4.14 / 4.16
	σ_{RhP} (*trans*) $\rightarrow \sigma^*_{CH}$	1.36 / 1.29
**[3][BAr$^F_4$]**	$\sigma_{CH} \rightarrow \sigma^*_{RhP}$ (*trans*)	11.43 / 12.62
	$\sigma_{CH} \rightarrow \sigma^*_{RhP}$ (*cis*)	0.07 / <0.05
	$LP_{Rh} \rightarrow \sigma^*_{CH}$	2.42 / 2.57
	σ_{RhP} (*cis*) $\rightarrow \sigma^*_{CH}$	2.62 / 2.22
	σ_{RhP} (*trans*) $\rightarrow \sigma^*_{CH}$	1.18 / 1.18

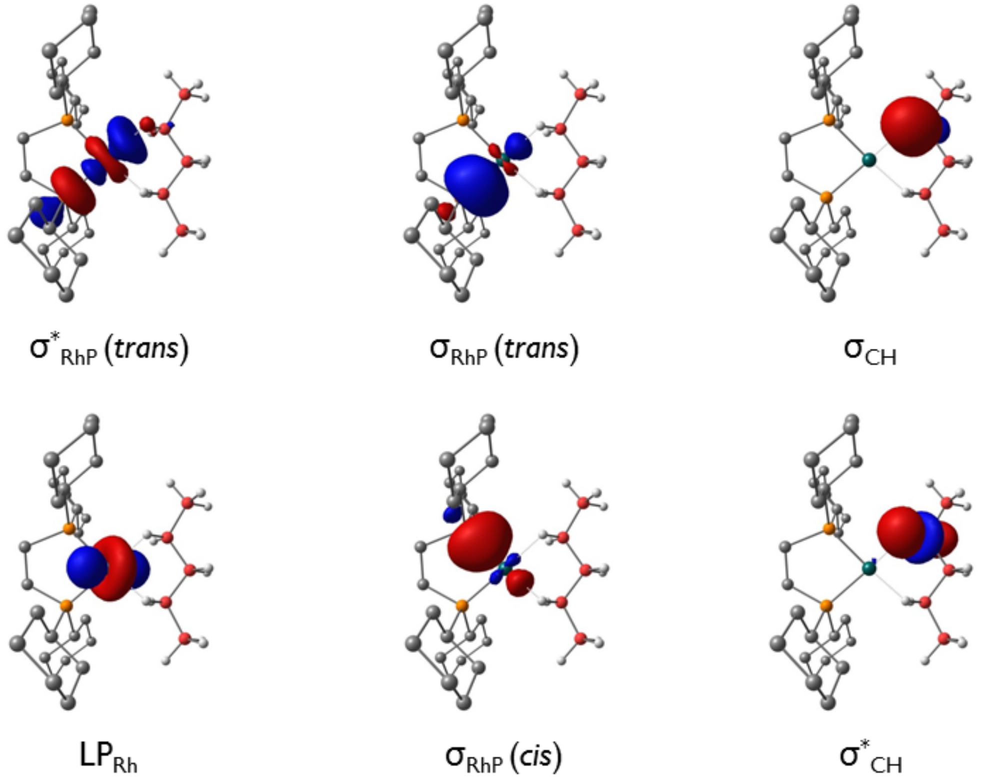

Figure S16: Important natural bond orbitals (NBOs) involved in the donor/acceptor bonding interaction in the cationic unit of **[3][BAr$^F_4$]** (*top view*).Carbon atoms of the pentane fragment in red.

10. Energies

Table S6: Relative energies of selected bond isomers of pentane in **[3][BAr$^F_4$]**.

Isomer	ΔE / kcal mol^{-1}
2,4-pentane	0.0
1,2-pentane	+17.3
1,3-pentane	+9.3
1,5-pentane	+13.7
2,3-pentane	+6.9
2,5-pentane	+6.0

11. Calculated $^1H/^{13}C$ NMR shifts

The GIPAW calculations on **[3][BAr$^F_4$]** utilized both the fully optimized and the refined crystal structure, in which only hydrogen atoms were relaxed during geometry optimisations while all other atoms were fixed on their crystallographic positions. Correlation plots for obtaining linear regression parameters (σ_{ref} and slope m) for both geometries are shown in Figure S14. In the plot for the fully optimized geometry one outlier can be identified, which corresponds to carbon in the CF_3 groups. A comparison between the fully optimized and crystal structure reveals that C–F bond distances are overestimated by ~0.05 Å in the fully optimised geometry, which in turn results in a decrease of the (averaged) magnetic shielding constant by ~15 ppm relative to the one obtained for the partially relaxed structure. This outlier was therefore omitted in the linear fit for this geometry. The markedly reduced σ_{iso} value naturally results in an overestimation of the associated chemical shift $\delta(^{13}C)$ for the CF_3 group. Nonetheless, we note that both geometries yield quantitatively similar results for the $^{13}C/^1H$ chemical shifts for the remaining parts of the molecule (Table S7). Due to the presence of (static) disorder in the crystal structure of **[2][BAr$^F_4$]** the geometry was fully optimised and subsequently used for GIPAW calculations. Like in the pentane complex **[3][BAr$^F_4$]**, the CF_3 carbon yields an outlier for the calculated chemical shielding and hence is omitted from the linear regression. A partially optimised geometry yielded unreasonable fitting parameters.

A

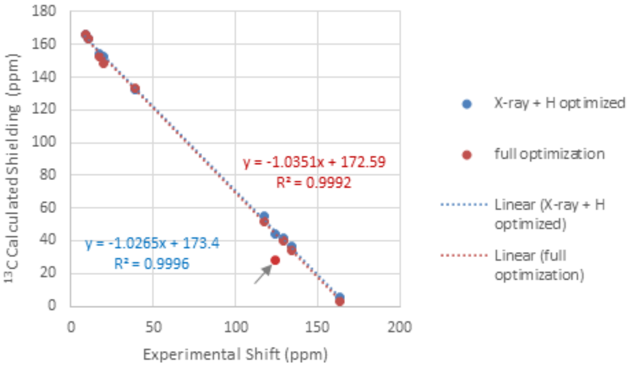

B

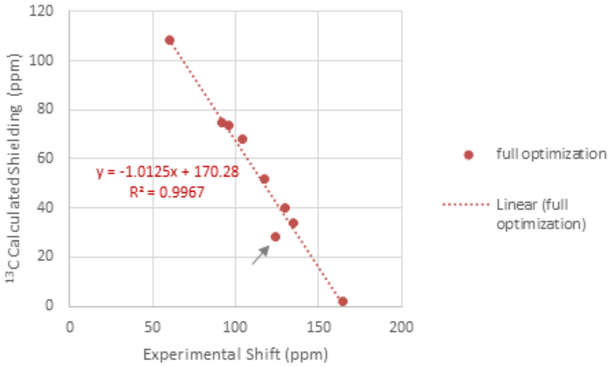

Figure S17: Correlation between calculated isotropic ^{13}C magnetic shieldings and experimental shifts for (**A**) **[3][BAr$^F_4$]** and (**B**) **[2][BAr$^F_4$]**. The arrow indicates an outlier in the fit using the fully optimized geometry, corresponding to the CF_3 group. This data point was not included in the linear regression.

Table S7: Comparison of ^{13}C and ^{1}H calculated chemical shifts obtained from using the fully optimized geometry and refined crystal structure of complex **[3][BAr$^F_4$]**.

	$\delta(^{13}C)$ / ppm			$\delta(^{1}H)$ / ppm	
	X-ray + H optimized	Full optimization		X-ray + H optimized	Full optimization
ipso-C	163.97	163.92	CH_3 (C1)	0.46 (avg)	0.51 (avg)
ortho-C	133.47	133.98	CH_2 (C2)	-1.60	-1.47
para-C	115.39	117.04		0.38	0.40
meta-C	128.14	128.05	CH_2 (C3)	-2.42	-2.52
CF_3	125.68	139.09		-0.85	-0.88
C1	12.33	11.88	CH_2 (C4)	-2.47	-2.63
C2	7.33	6.15		0.74	0.74
C3	40.13	37.82	CH_3 (C5)	0.58 (avg)	0.60 (avg)
C4	9.89	9.03			
C5	12.95	12.01			

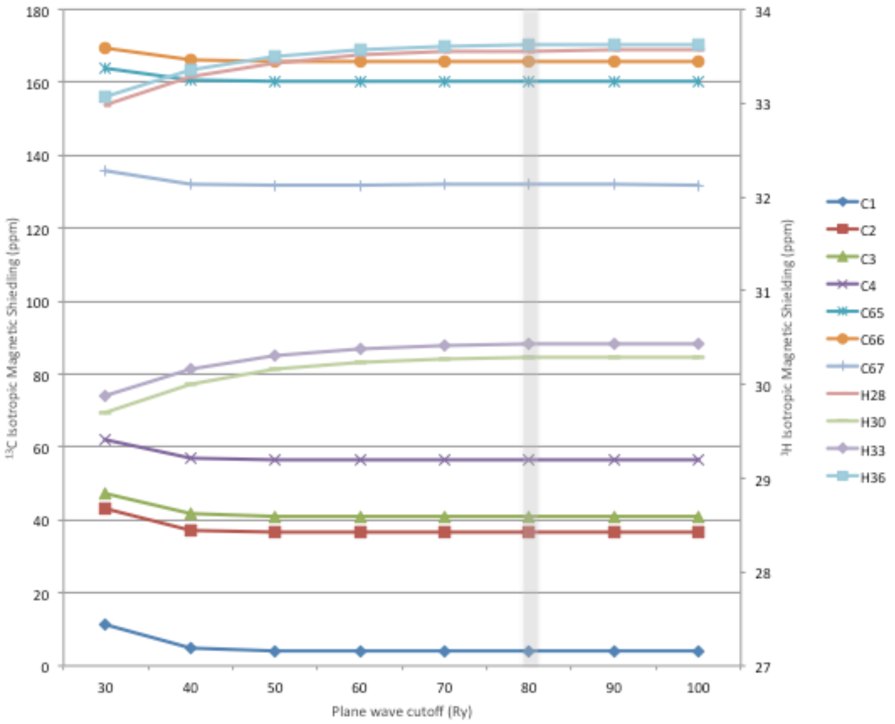

Figure S18. Convergence of isotropic magnetic shielding with respect to plane wave cutoff.

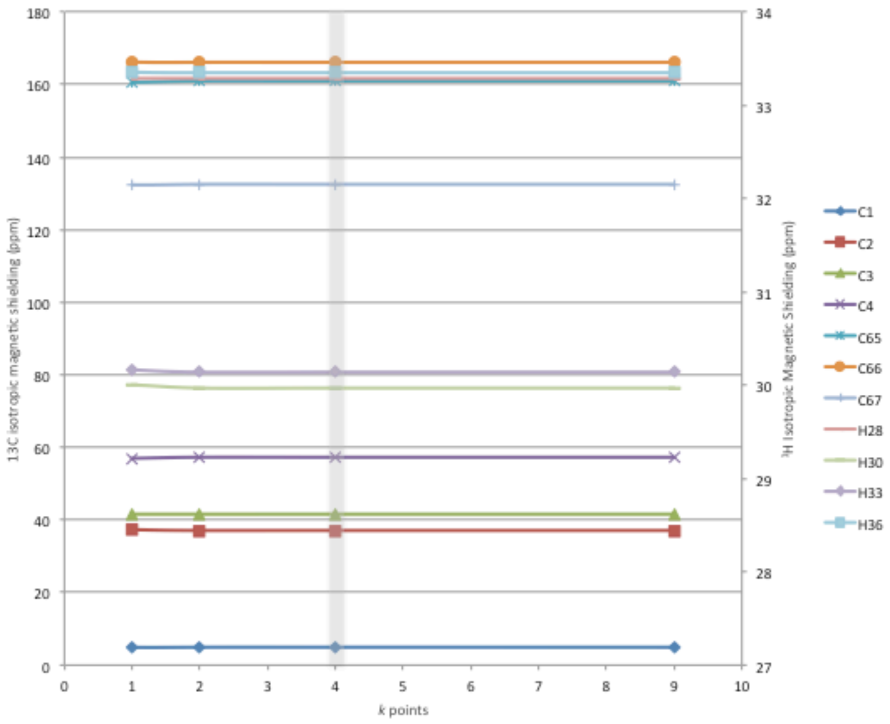

Figure S19. Convergence of isotropic magnetic shielding constants with respect to *k*-point sampling.

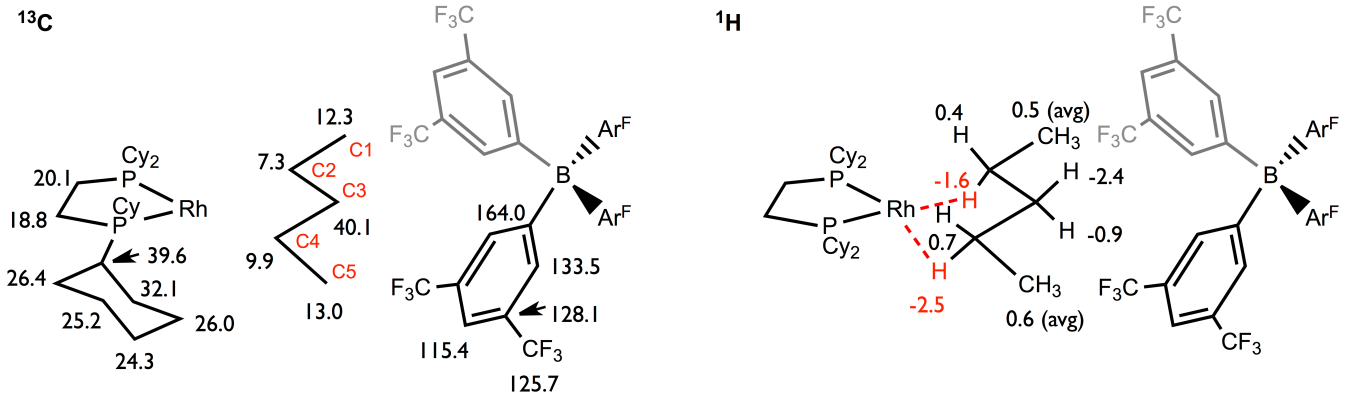

Figure S20. Calculated (GIPAW, PBE, planewave cutoff 80 Ry) NMR chemical shifts, $\delta(^{13}C)$ and $\delta(^{1}H)$ for **[3][BAr$^F_4$]**.

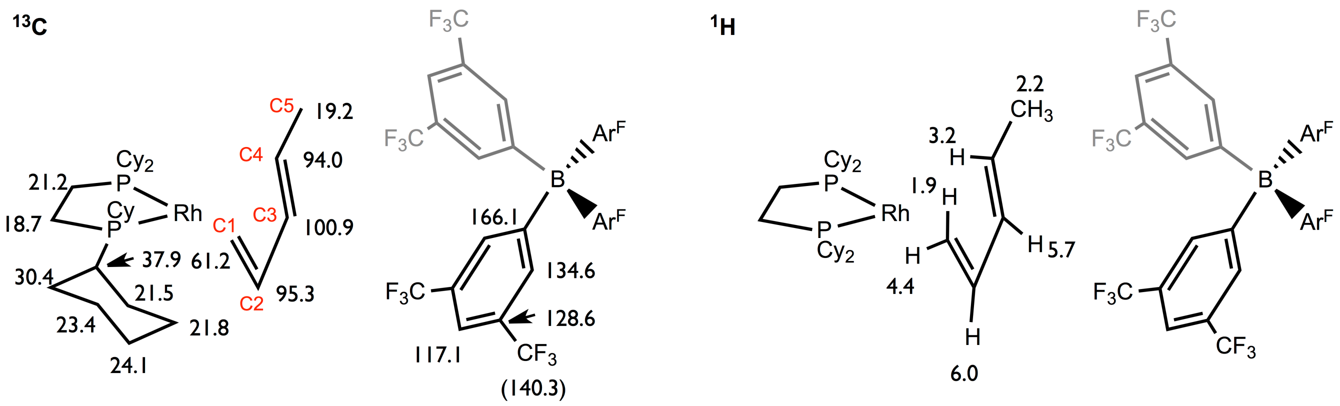

Figure S21. Calculated (GIPAW, PBE, planewave cutoff 80 Ry) NMR chemical shifts, $\delta(^{13}C)$ and $\delta(^{1}H)$ for **[2][BAr$^{F}_{4}$]**.

12. Further discussion of metadynamics

All the three runs show frequent torsional rotations and changes in coordination mode of the addressed pentane molecule, whereas the other pentane molecule in the simulation cell remains unaffected. The transformations bring the initial structure, where pentane is coordinated via the C2 and C4 groups to Rh, to another metastable state, where the coordination occurs via C1 and C3. Between these two minima, intermediate states are visited, which correspond to a large variety of geometries with torsion angle smaller than 180 deg. All these structures are clearly higher in potential energy. However, the increased degree of disorder and the large variety of possible configurations enhance the statistical probability of the transition region , which behaves as an entropic bottleneck and traps the structure into an unstable situation. These effects seem to be even more enhanced by increasing the temperature, as appearing from the 2D projection of the FES obtained at the three considered temperatures. These results point to a flexibility of the cavity containing pentane, which becomes more significant at higher temperatures.

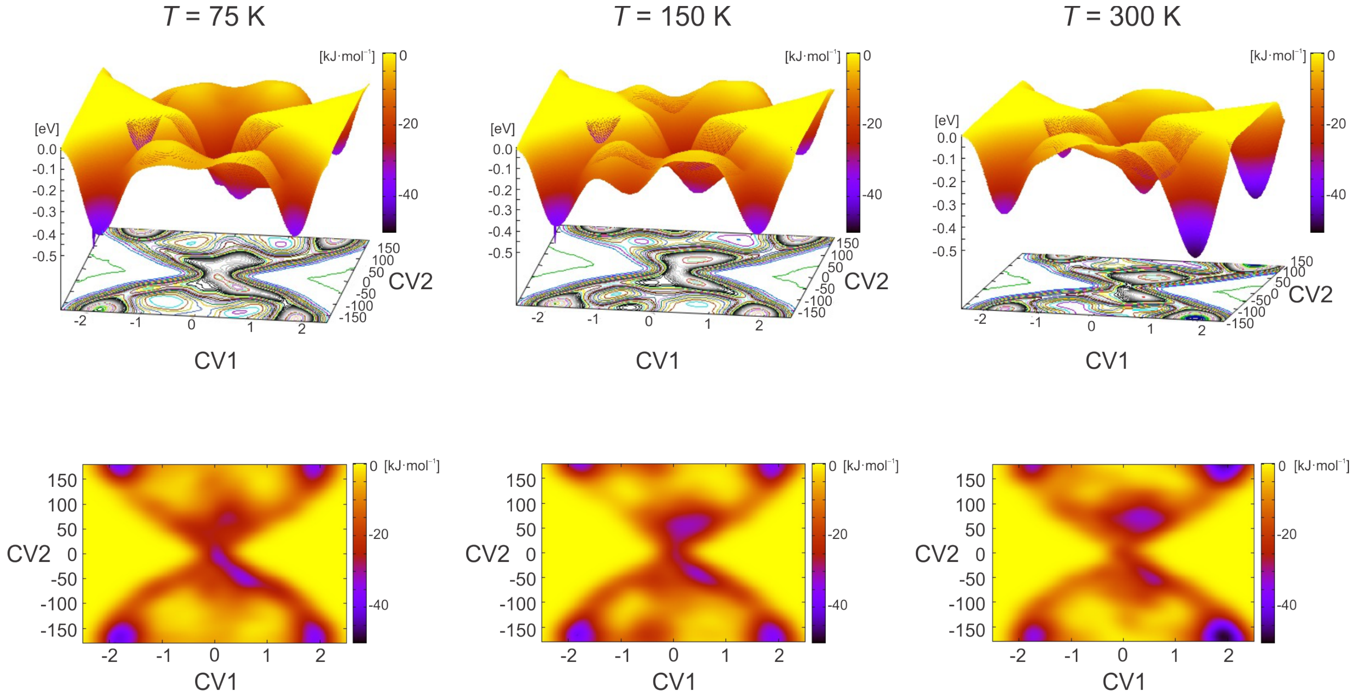

Figure S22. Free energy surfaces from MTD simulations at $T = 75$, 150 and 300 K for **[3][BAr$^F_4$]**.

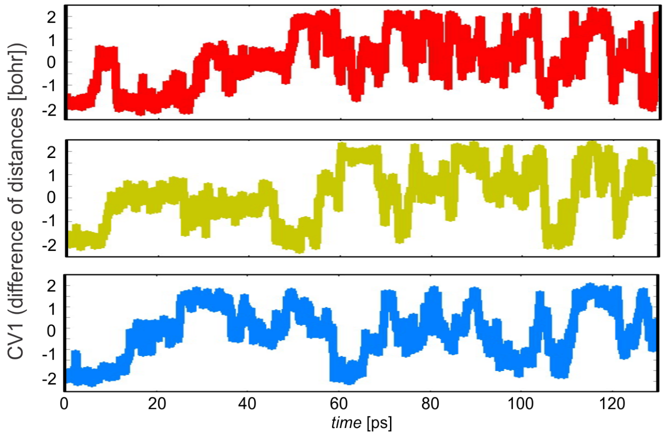

Figure S23. Time evolution of collective variable CV1 during simulation ($t \approx 130$ ps) at $T = 75$, 150 and 300 K for **[3][BAr$^F_4$]**.

13. References

[1] O. B. Peersen, X. L. Wu, I. Kustanovich, S. O. Smith, *J. Magn. Reson. Ser. A* **1993**, *104*, 334–339.
[2] W. L. Earl, D. . Vanderhart, *J. Magn. Reson.* **1982**, *48*, 35–54.
[3] B.-J. van Rossum, H. Förster, H. J. M. de Groot, *J. Magn. Reson.* **1997**, *124*, 516–519.
[4] A. T. Lubben, J. S. McIndoe, A. S. Weller, *Organometallics* **2008**, *27*, 3303–3306.
[5] J. Cosier, A. M. Glazer, *J. Appl. Crystallogr.* **1986**, *19*, 105–107.
[6] Oxford Diffraction Ltd., **2011**.
[7] A. Altomare, G. Cascarano, C. Giacovazzo, A. Guagliardi, M. C. Burla, G. Polidori, M. Camalli, *J. Appl. Crystallogr.* **1994**, *27*, 435–435.
[8] L. Palatinus, G. Chapuis, *J. Appl. Crystallogr.* **2007**, *40*, 786–790.
[9] P. W. Betteridge, J. R. Carruthers, R. I. Cooper, K. Prout, D. J. Watkin, *J. Appl. Crystallogr.* **2003**, *36*, 1487–1487.
[10] R. I. Cooper, A. L. Thompson, D. J. Watkin, *J. Appl. Crystallogr.* **2010**, *43*, 1100–1107.
[11] W. E. Buschmann, J. S. Miller, *Inorg. Synth.* **2002**, *33*, 83–91.
[12] S. D. Pike, F. M. Chadwick, N. H. Rees, M. P. Scott, A. S. Weller, T. Krämer, S. A. Macgregor, *J. Am. Chem. Soc.* **2014**, *137*, 820–833.
[14] VandeVondele, J.; Krack, M.; Mohamed, F.; Parrinello, M.; Chassaing, T.; Hutter, J. *Comput Phys Commun* **2005**, *167*, 103.
[15] (a) The CP2K developers group, http://www.cp2k.org(b) Hutter, J.; Iannuzzi, M.; Schiffmann, F.; VandeVondele, J. *Wires Comput Mol Sci* **2014**, *4*, 15.
[16] VandeVondele, J.; Hutter, J. *J Chem Phys* **2007**, *127*, 114105.
[17] (a) Hartwigsen, C.; Goedecker, S.; Hutter, J. *Phys Rev B* **1998**, *58*, 3641(b) Goedecker, S.; Teter, M.; Hutter, J. *Phys Rev B* **1996**, *54*, 1703(c) Krack, M. *Theor Chem Acc* **2005**, *114*, 145.
[18] Perdew, J. P.; Burke, K.; Ernzerhof, M. *Phys Rev Lett* **1996**, *77*, 3865.
[19] Grimme, S.; Antony, J.; Ehrlich, S.; Krieg, H. *J Chem Phys* **2010**, *132*, 154104.
[20] Marx, D.; Hutter, J. *Ab Initio Molecular Dynamics*; Cambridge University Press, 2009.
[21] Laio, A.; Parrinello, M. *P Natl Acad Sci USA* **2002**, *99*, 12562.
[22] Iannuzzi, M.; Laio, A.; Parrinello, M. *Phys Rev Lett* **2003**, *90*, 238302.
[23] Gaussian 09 (Revision D.01); Frisch, M. J.; Trucks, G. W.; Schlegel, H. B.; Scuseria, G. E.; Robb, M. A.; Cheeseman, J. R.; Scalmani, G.; Barone, V.; Mennucci, B.; Petersson, G. A.; Nakatsuji, H.; Caricato, M.; Li, X.; Hratchian, H. P.; Izmaylov, A. F.; Bloino, J.; Zheng, G.; Sonnenberg, J. L.; Hada, M.; Ehara, M.; Toyota, K.; Fukuda, R.; Hasegawa, J.; Ishida, M.; Nakajima, T.; Honda, Y.; Kitao, O.; Nakai, H.; Vreven, T.; J. A. Montgomery, J.; Peralta, J. E.; Ogliaro, F.; Bearpark, M.; Heyd, J. J.; Brothers, E.; Kudin, K. N.; Staroverov, V. N.; Keith, T.; Kobayashi, R.; Normand, J.; Raghavachari, K.; Rendell, A.; Burant, J. C.; Iyengar, S. S.; Tomasi, J.; Cossi, M.; Rega, N.; Millam, J. M.; Klene, M.; Knox, J. E.; Cross, J. B.; Bakken, V.; Adamo, C.; Jaramillo, J.; Gomperts, R.; Stratmann, R. E.; Yazyev, O.; Austin, A. J.; Cammi, R.; Pomelli, C.; Ochterski, J. W.; Martin, R. L.; Morokuma, K.; Zakrzewski, V. G.; Voth, G. A.; Salvador, P.; Dannenberg, J. J.; Dapprich, S.; Daniels, A. D.; Farkas, O.; Foresman, J. B.; Ortiz, J. V.; Cioslowski, J.; Fox, D. J.; Gaussian Inc., Wallingford, CT, 2013.
[24] (a) Becke, A. D. *Phys Rev A* **1988**, *38*, 3098(b) Perdew, J. P. *Phys Rev B* **1986**, *33*, 8822.
[25] Andrae, D.; Haussermann, U.; Dolg, M.; Stoll, H.; Preuss, H. *Theor Chim Acta* **1990**, *77*, 123.
[26] Hollwarth, A.; Bohme, M.; Dapprich, S.; Ehlers, A. W.; Gobbi, A.; Jonas, V.; Kohler, K. F.; Stegmann, R.; Veldkamp, A.; Frenking, G. *Chem Phys Lett* **1993**, *208*, 237.
[27] (a) Hehre, W. J.; Ditchfie.R; Pople, J. A. *J Chem Phys* **1972**, *56*, 2257(b) Harihara, P. C.; Pople, J. A. *Theor Chim Acta* **1973**, *28*, 213.
[28] Bader, R. F. W. *Atoms in Molecules: A Quantum Theory*; Oxford University Press, 1990.
[29] AIMAll (Version 13.02.26), Todd A. Keith, TK Gristmill Software, Overland Park KS, USA, 2014 (aim.tkgristmill.com) ed., 1997-2013.
[30] NBO5.9; Glendening, E. D. *et al.*; (Theoretical Chemistry Insitute, Univ. of Wisconsin, Madison, WI); www.chem.wis.edu/~nbo5 ed., 2009.

[31] Johnson, E. R.; Keinan, S.; Mori-Sanchez, P.; Contreras-Garcia, J.; Cohen, A. J.; Yang, W. T. *J Am Chem Soc* **2010**, *132*, 6498.

[32] (a) Pickard, C. J.; Mauri, F. *Phys Rev B* **2001**, *63*, 245101(b) Yates, J. R.; Pickard, C. J.; Mauri, F. *Phys Rev B* **2007**, *76*, 024401.

[33] Clark, S. J.; Segall, M. D.; Pickard, C. J.; Hasnip, P. J.; Probert, M. J.; Refson, K.; Payne, M. C. *Z Kristallogr* **2005**, *220*, 567.

[34] Monkhorst, H. J.; Pack, J. D. *Phys Rev B* **1976**, *13*, 5188.

[35] Lodewyk, M. W.; Siebert, M. R.; Tantillo, D. J. *Chem Rev* **2012**, *112*, 1839.
